# Nuclear receptor NR5A2 controls neural stem cell fate decisions during development

**DOI:** 10.1038/ncomms12230

**Published:** 2016-07-22

**Authors:** Athanasios Stergiopoulos, Panagiotis K. Politis

**Affiliations:** 1 Center for Basic Research, Biomedical Research Foundation of the Academy of Athens, 4 Soranou Efesiou Street, 115 27 Athens, Greece

## Abstract

The enormous complexity of mammalian central nervous system (CNS) is generated by highly synchronized actions of diverse factors and signalling molecules in neural stem/progenitor cells (NSCs). However, the molecular mechanisms that integrate extrinsic and intrinsic signals to control proliferation versus differentiation decisions of NSCs are not well-understood. Here we identify nuclear receptor NR5A2 as a central node in these regulatory networks and key player in neural development. Overexpression and loss-of-function experiments in primary NSCs and mouse embryos suggest that NR5A2 synchronizes cell-cycle exit with induction of neurogenesis and inhibition of astrogliogenesis by direct regulatory effects on *Ink4*/*Arf* locus, *Prox1*, a downstream target of proneural genes, as well as Notch1 and JAK/STAT signalling pathways. Upstream of *NR5a2*, proneural genes, as well as Notch1 and JAK/STAT pathways control *NR5a2* endogenous expression. Collectively, these observations render NR5A2 a critical regulator of neural development and target gene for NSC-based treatments of CNS-related diseases.

## Abstract (web summary)

The molecular signals regulating the decision of neural stem cells (NSC) to proliferate versus differentiate are unclear. Here, the authors identify the nuclear receptor NR5A2 as coordinating cell-cycle exit with differentiation of NSCs via direct actions on Ink4, Prox1, Notch1 and JAK/STAT.

## Graphical abstract

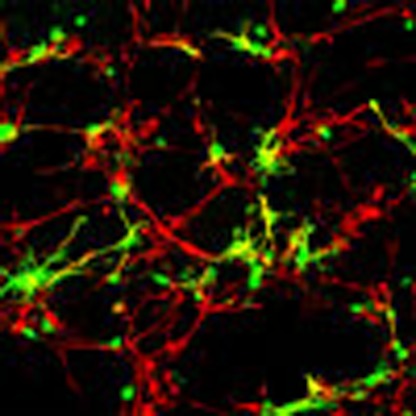

The question of how the enormous complexity of central nervous system (CNS) is generated during mammalian development is still open^1^. Identification of novel players in the control of cell-cycle exit and differentiation of neural stem/progenitor cells (NSCs), may not only allow new therapeutic approaches, but also provide mechanistic insights in nervous system formation and function.

In this regard, here we report for the first time the identification of nuclear receptor NR5A2 (also known as LRH1) as a key node in the gene regulatory networks that control NSC differentiation. NR5A2 is a constitutively active orphan nuclear receptor with well-established, FDA-approved, pharmacological agonists/antagonists^2^,^3^,^4^,^5^. Moreover, NR5A2 has important roles in early embryogenesis since knockout mice die between *E6.5* and *E7.5* (refs 6, 7, 8), maintenance of pluripotency in embryonic stem cells (ESCs)^6^ and reprogramming of somatic cells into iPSCs^9^,^10^. Later on during organogenesis and tissue homeostasis, NR5A2 is involved in promoting proliferation of progenitors and cancer cells from various tissues^11^,^12^,^13^,^14^,^15^. Despite these effects, NR5A2 has not been previously correlated with nervous system development or function. Most importantly, these data raised the hypothesis that NR5A2 may be able to promote proliferation and expansion of NSC pool during brain and spinal cord organogenesis.

In contrast, we show here that NR5A2 blocks proliferation of NSCs and additionally induces neuronal differentiation via distinct mechanistic actions. In particular, NR5A2 controls proliferation and differentiation of NSCs via direct regulatory effects on *Ink4*/*Arf* locus (*p16*^*Ink4a*^ and *p15*^*Ink4b*^ genes), *Prox1* gene, as well as Notch1 and JAK/STAT pathways. Interestingly, *NR5a2* is upstream regulated by the major neuronal and astrocytic pathways including proneural genes (*Neurog2* and *Mash1*), as well as Notch1 and JAK/STAT signalling. Overall, we describe a new mechanism in the regulation of neurogenesis during CNS development that may allow novel approaches in neuro-regeneration.

## Results

### NR5A2 expression is associated with the neuronal lineage

**Figure 1:**
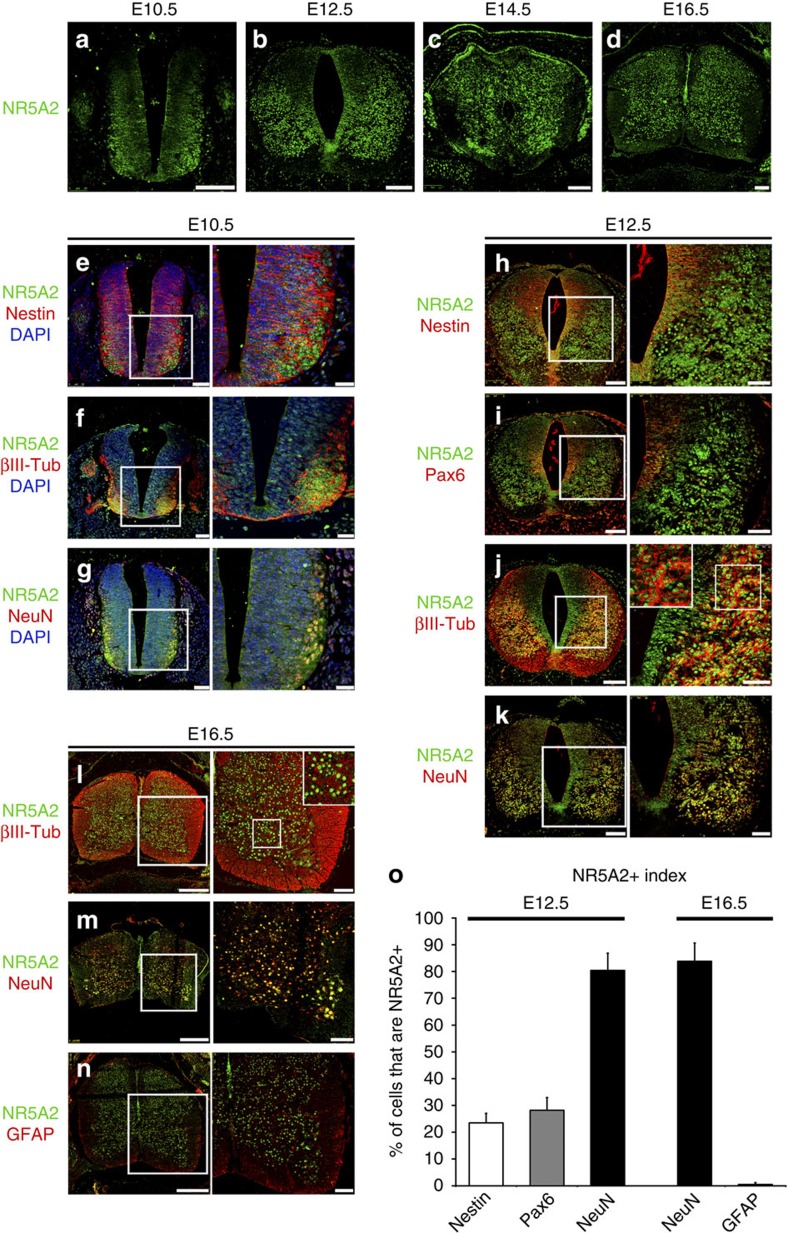
NR5A2 expression pattern is correlated with neuronal lineage during spinal cord development. (**a**–**d**) Immunostainings of NR5A2 (green) on transverse paraffin sections from *E10.5* (**a**), *E12.5* (**b**), *E14.5* (**c**) or *E16.5* (**d**) mouse spinal cord. (**e**–**n**) Double immunostainings of NR5A2 (green) with Nestin (**e**, **h**), Pax6 (**i**), βIII-Tubulin (**f**, **j**, **l**), NeuN (**g**, **k**, **m**) or GFAP (**n**) (all red) at *E10.5* (**e**–**g**), *E12.5* (**h**–**k**) or *E16.5* (**l**–**n**) mouse spinal cord, as indicated. Control immunofluorescences without primary antibody showed no staining. Inlets in the right panels of **j** and **l** represent larger magnifications of the areas included into the square shapes of the corresponding images. (**o**) Quantification of the cell populations that express NR5A2 (% of marker+; NR5A2+/total marker+). Values represent the mean±s.d. of four animals (*n=*4). Scale bars, (**a**–**d**), 100 μm; (**e**–**g**), 50 μm, 25 μm; (**h**–**k**), 100 μm, 50 μm; (**l**–**n**), 250 μm, 75 μm.

To investigate the involvement of NR5A2 in neural development, we first examined its developmental expression pattern (Fig. 1a–d). Surprisingly enough, NR5A2 was detected in high levels in βIII-Tubulin+ and NeuN+ neurons of the mantle zone (MZ) in early neural tube (*E10.5* and *E12.5*) and, more weakly, in a subset of Nestin+ NSCs or Pax6+ neuronal progenitors of the ventricular zone (VZ) (Fig. 1e–k,o). Consistent with a correlation with neuronal differentiation, a larger number of NR5A2+ cells was detected in spinal cord at later developmental stages, *E12.5* or *E16.5*, as compared with earlier stages, that is, *E10.5* (Fig. 1e–o). Moreover, a medio-lateral gradient of NR5A2 expression was also evident, with lower NR5A2 levels in the neuroepithelial VZ cells and higher in the differentiated neuronal cells of the MZ. Interestingly, NR5A2 expression was totally excluded from GFAP+ astrocytes (Fig. 1n,o). Similar expression pattern was observed in the developing telencephalon, where NR5A2 levels were higher in the βIII-Tubulin+ and NeuN+ neurons of the MZ than Nestin+ and Pax6+ cells of the VZ (Supplementary Fig. 1a–k).

We further confirmed these observations by double immunostainings in acute dissociated cells (6 h after trituration) and *ex vivo*-cultured NSCs, both derived from spinal cords of *E14.5* mouse embryo. In these cells, NR5A2 is expressed at much higher levels in βIII-Tubulin+ neurons compared with Nestin+ NSCs or Pax6+ neural precursors and much lower in BrdU+ (after 2-h pulse) proliferating NSCs (Supplementary Fig. 1l–n,p–r,t). Similar to the *in vivo* situation, NR5A2 is not detected in GFAP+ astrocytes (Supplementary Fig. 1o,s,t). These data suggest that NR5A2 is initially expressed in neuronal progenitor cells (Nestin+, Pax6+), induced in the transition of these cells towards the post-mitotic neuronal identity (βIII-Tubulin+ and NeuN+), while excluded from astrocytes (GFAP+).

### NR5A2 is differentially regulated by upstream signals

**Figure 2:**
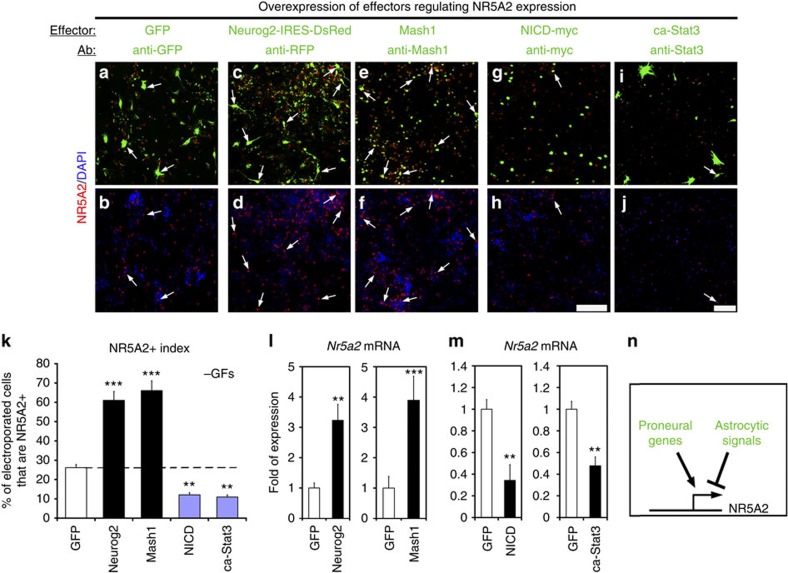
NR5A2 expression is regulated by proneural and astrogliogenic signals. (**a**–**j**) Double GFP/NR5A2 (**a**,**b**), RFP (Neurog2, pseudo-colored green)/NR5A2 (**c**,**d**), Mash1/NR5A2 (**e**,**f**), Myc(NICD)/NR5A2 (**g**,**h**) or Stat3/NR5A2 (**i**,**j**) immunostainings of NSCs electroporated with various constructs, as indicated. Arrows mark representative cells that co-express the transgenes (green) and NR5A2 protein (red). Cell nuclei were visualized with DAPI staining (blue). (**k**) Quantification of electroporation data shown in **a**–**j** (% of transgene+; NR5A2+/total transgene+). (**l**,**m**) RT–qPCRs showing the quantification of *Nr5a2* mRNA levels in differentiating NSCs, electroporated with GFP, Neurog2, Mash1, NICD-myc and ca-Stat3, as indicated. (**n**) Schematic representation of the upstream signals that regulate NR5A2. In every case, arrows indicate representative electroported cells that co-express each marker. The results are shown as mean±s.d. **P<0.01, ***P<0.001 (Student's *t*-test). Scale bars, 100 μm.

Consistently, overexpression of proneural genes, such as Mash1 and Neurog2, potent inducers of neuronal differentiation^1^,^16^,^17^, strongly upregulates the expression of *Nr5a2* (Fig. 2a–f,k–l,n). On the other hand, induction of well-established astrogliogenic and proliferative signals in *ex vivo*-cultured NSCs, reduces the expression of *Nr5a2*. In particular, the overexpression of constitutively active Notch1 (NICD, Notch1 intracellular domain) or Stat3 (ca-Stat3) leads to downregulation of *Nr5a2* (Fig. 2g–k,m,n). Both Notch1 and Stat3 pathways induce self-renewal and astrogliogenesis^18^,^19^,^20^, suggesting that NR5A2 has to be repressed to allow NSC proliferation and differentiation into astrocytes. However, an interesting question is whether NR5A2 is only a marker for neuronal identity or an important target of these pathways and therefore critical regulator for the acquisition of neuronal fate.

### NR5A2 controls proliferation versus differentiation of NSCs

**Figure 3:**
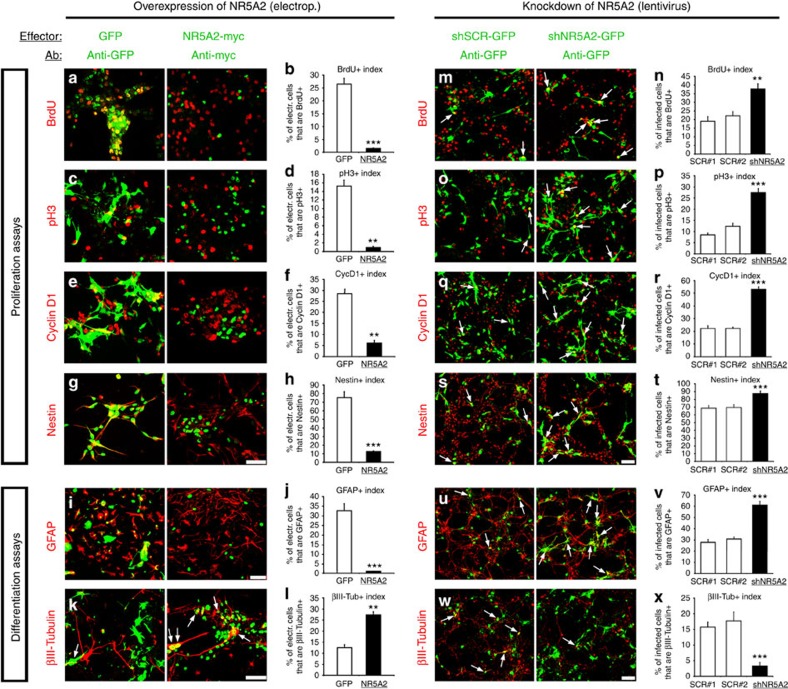
NR5A2 regulates self-renewal and differentiation properties of NSCs. (**a**–**l**) Double immunostainings of Amaxa-electroporated NSCs with GFP or NR5A2-myc (green, detected with myc) and various markers (all red), as indicated (**a**–**k**). Quantifications of the indices of the above markers are shown in **b**, **d**, **f**, **h**, **j** and **l**, respectively (% of GFP+ or NR5A2-myc+; marker+/total GFP+ or NR5A2-myc+). (**m**–**x**) Double immunostainings of GFP with the same markers, as indicated. Cells were infected with lentiviruses expressing control-scrambled sequences (shSCR#1-GFP, shSCR#2-GFP) or one potent shRNA targeting mouse NR5A2 (shNR5A2-GFP). All vectors co-express GFP from independent promoters. Quantifications of the indices are shown in **n**, **p**, **r**, **t**, **v** and **x** (% of GFP+; marker+/total GFP+). In every case, arrows indicate representative GFP+ or NR5A2-myc+ cells that co-express each marker. The results are shown as mean±s.d. **P<0.01, ***P<0.001 (Student's *t*-test). Scale bars, 50 μm.

To initially investigate this hypothesis, we performed overexpression and knockdown experiments in *ex vivo*-cultured NSCs (Supplementary Fig. 2a). Adenoviral-mediated overexpression of NR5A2 strongly reduced the ability of these cells to form spheres in free-floating conditions (+GFs) (Supplementary Fig. 2b–f), with no indication of increased cell death (active Caspase 3) (Supplementary Fig. 2g,h). In addition, BrdU incorporation analysis 72 h after Amaxa electroporation of NR5A2 expression plasmid, followed by 2-h BrdU pulse, revealed a strong reduction in BrdU incorporation by 94.2% in a cell–autonomous manner as compared with GFP electroporation (Fig. 3a,b). Similarly, dramatic decreases by 94.0% in the proportion of phosphorylated Histone H3+ (pH3), 78.5% in CyclinD1+ and 83.3% in Nestin+ (Fig. 3c–h) cells were specifically observed in NR5A2-transfected cells. Moreover, real time reverse transcription–quantitative PCR (RT–qPCR) analyses indicated that adenoviral-mediated overexpression of NR5A2 reduced the expression of *Ki67*, *Nestin* and *CyclinD1* genes (Supplementary Fig. 3a). Therefore, all these indices and mRNA analyses show that NR5A2 negatively affects self-renewal and proliferation of NSCs. We next asked whether NR5A2 overexpression also influences astrogliogenesis and neurogenesis. Under differentiation conditions (-GFs), generation of GFAP+ astrocytes was severely impaired (by 96.7%) in the NR5A2-electroporated NSCs (Fig. 3i,j). On the other hand, a significant increase (Student's *t*-test) in the proportion of βIII-Tubulin+ neurons by ∼2.5-fold was observed (Fig. 3k,l). In agreement, by real time RT–qPCR, we showed that overexpression of NR5A2 reduced the expression of astrogliogenesis-related genes, including *Gfap*, *Nfia, Nfib* and *S100β*, and induced neurogenesis-related genes, including *NeuN* and *Map2* (Supplementary Fig. 3).

Conversely, lentiviral-mediated shRNA knockdown of NR5A2 (Supplementary Fig. 4a–j) caused a significant increase (Student's *t*-test) in the proliferation indices, including BrdU incorporation, pH3+, CyclinD1+ and Nestin+ (Fig. 3m–t) cells. Remarkably, under conditions of NR5A2 knockdown, we were able to measure a substantial number of double BrdU+/pH3+cells that we were not able to score with two independent shControl lentiviral vectors (Supplementary Fig. 4k–m), indicating a population of shNR5A2+ NSCs with much faster cell-cycle properties. In agreement, shRNA for NR5A2 was sufficient to induce the number of Nestin+ NSCs even under differentiation conditions (Supplementary Fig. 4n,o). Moreover, NR5A2 downregulation led to enhanced numbers of GFAP+ astrocytes (Fig. 3u,v). Most importantly, neuronal differentiation was significantly impaired (Student's *t*-test), as evidenced by measuring the index of βIII-Tubulin+ NSC-derived neurons (Fig. 3w,x), without inducing an apoptotic response (Supplementary Fig. 4p,q). In addition, by real time RT–qPCR, we showed that knockdown of NR5A2 induces the expression of proliferation-related genes (*Ki67*, *Nestin*, *CyclinD1* and *CyclinE1*) and astrogliogenesis-related genes (*Gfap*, *Nfia*, *Nfib* and *S100β*), whereas reduces the expression of neurogenesis-related genes (*NeuN*, *Map2*, *Dcx* and *βIII-Tubulin*). (Supplementary Fig. 3d,f). Collectively, our results demonstrate that NR5A2 is sufficient and necessary for negative regulation of self-renewal properties of NSCs, initiation of neuronal differentiation and suppression of astrogliogenesis.

### Inducible deletion of *Nr5a2* affects neural development

**Figure 4:**
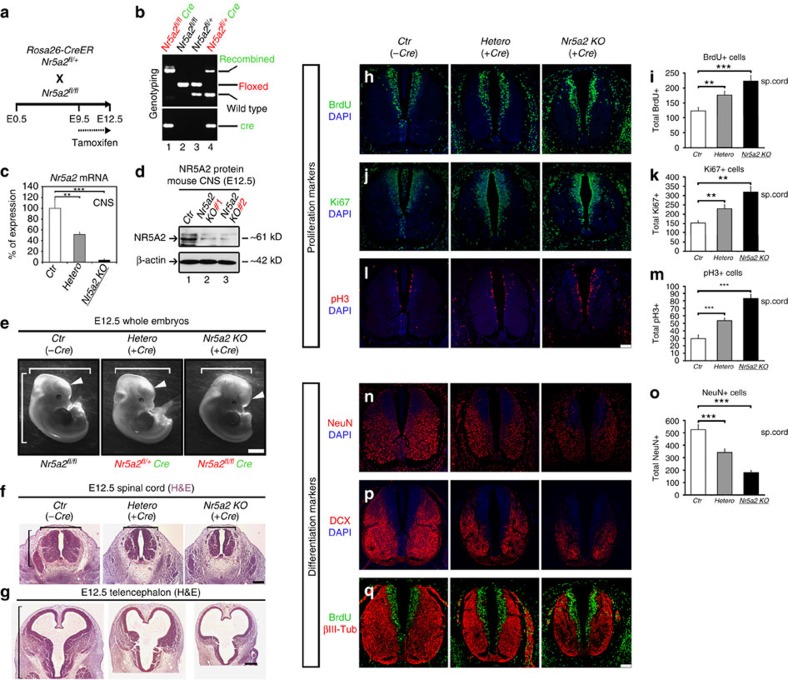
Temporal deletion of NR5A2 affects mouse CNS development. (**a**) Schematic representation of the knockout strategy. (**b**) Assessment of recombination of the floxed *Nr5a2* locus by genotyping PCR in CNS tissue, as indicated. Note the efficient recombination of the floxed *Nr5a2* alleles in the presence of a single allele of *CreER* (lane 1). (**c**,**d**) RT–qPCR (**c**) and western blot (**d**) analyses in *E12.5* CNS of *Ctr*, *Hetero* and *Nr5a2 KO* animals for the detection of *Nr5a2* mRNA and protein levels, respectively. (**e**) Stereoscopic views of *E12.5 Ctr*, *Hetero* and *Nr5a2 KO* whole-mouse embryos. The white arrowheads depict the forebrain of each embryo. Note the large difference in the size of the forebrain of *Nr5a2 KO* mice (right panel). (**f**–**g**) Histological sections of *E12.5* spinal cords (**f**) and *E12.5* telencephalons (**g**) of *Ctr*, *Hetero* and *Nr5a2 KO* littermates, stained with haematoxylin & eosin. Note the substantial size reduction of both structures (square brackets) in *Nr5a2 KO* embryos and to a lesser extent in *Hetero* embryos. (**h**–**q**) Phenotypic analysis of *Hetero* (centre panels) and *Nr5a2 KO* (right panels) spinal cords, compared with *Ctr* (left panels), in relation to various proliferation (**h**–**m**) or differentiation (**n**–**q**) markers, as indicated. Note the ectopic proliferating cells (BrdU+, Ki67+, pH3+) that are localized outside the VZ and do not express neuronal markers (for example, βIII-Tubulin). Quantifications of BrdU+, Ki67+, pH3+ and NeuN+ cells are shown in **i**, **k**, **m** and **o**, respectively (total marker+/spinal cord section). All values represent the mean±s.d. of four animals (*n=*4). **P<0.01, ***P<0.001 (Student's *t*-test). Scale bars, (**e**) 250 μm; (**f**,**g**), 250 μm; (**h**–**q**), 75 μm.

**Figure 5:**
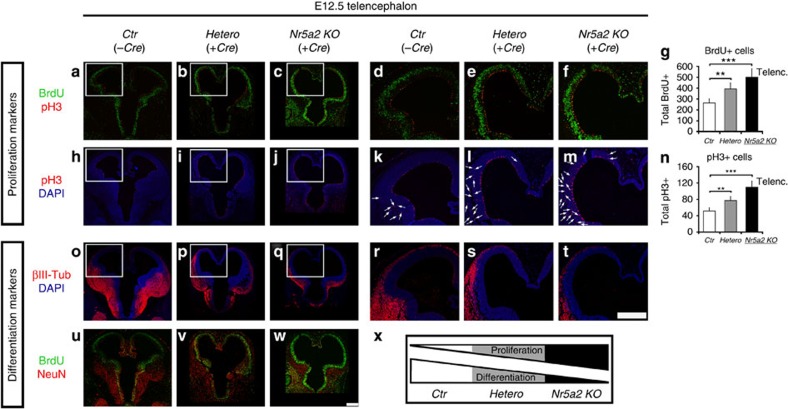
Cre/LoxP-mediated temporal deletion of NR5A2 affects telencephalon development. (**a**–**w**) Phenotypic analysis of *Hetero* (center panels) and *Nr5a2 KO* (right panels) experimental *E12.5* telencephalons, compared with *Ctr* (left panels), in relation to various proliferation (**a**–**n**) or differentiation (**o**–**w**) markers: immunofluorescence images of representative sections depicting the double BrdU+ (green)/pH3+ (red) (**a**–**f**), pH3+ (**h**–**m**), βIII-Tubulin+ (**o**–**t**) and BrdU+ (green)/NeuN+ (red) (**u**–**w**) cells. **d**–**f**, **k**–**m**, **r**–**t** micrographs are larger magnifications of the square shapes depicted in **a**–**c**, **h**–**j**, **o**–**q**, respectively. Quantifications of BrdU+ and pH3+ cells are shown in **g** and **n**, respectively (total BrdU+ or pH3+/telencephalon section). Arrows in representative pictures (**k**–**m**) indicate the ectopic pH3+-proliferating cells that are localized outside the VZ. (**x**) Schematic representation of the effects of NR5A2 deletion on proliferation and differentiation markers in the telencephalon of *E12.5* mouse embryos. All values represent the mean±s.d. of four animals (*n=*4). **P<0.01, ***P<0.001 (Student's *t*-test). Scale bars, 250 μm.

We next examined the *in vivo* requirement of NR5A2 for proper development of mouse CNS. The germline knockout of *Nr5a2* is lethal very early during mouse embryogenesis (*E6.5*–*E7.5*)^6^,^7^. To ablate NR5A2 expression in an inducible manner during later developmental stages in mouse CNS, we crossed the *Nr5a2* floxed (*Nr5a2*^*fl/fl*^)^11^,^12^,^21^ with *Rosa26-CreER* mice (Fig. 4a). In particular, we evaluated CNS development in *Nr5a2*^*fl/fl*^;*CreER* embryos (*Nr5a2 KO*), and compared them with their heterozygous *Nr5a2*^*fl/+*^;*CreER* (*Hetero*) and wild-type *Nr5a2*^*fl/fl*^*;without CreER*, littermates (*Ctr*). By applying tamoxifen to the pregnant mothers for 3 days (*E9.5*–*E12.5*) (Fig. 4a,b), the floxed alleles of *Nr5a2* gene were efficiently recombined in the CNS (Supplementary Fig. 5a,b), and *Nr5a2* mRNA and protein levels were sharply diminished (Fig. 4c,d), resulting in substantial reduction in the size of spinal cord and brain (Fig. 4e–g and Supplementary Fig. 6). Moreover, detailed immunohistochemical analysis revealed strong increase of proliferating cells (BrdU+, Ki67+ and pH3+ cells) (Fig. 4h–m, larger magnifications in Supplementary Fig. 7a–c) in the spinal cord of *Nr5a2 KO* embryos and to a lesser extent in *Hetero* embryos. Remarkably, many ectopically proliferating cells outside the VZ were evident in knockout spinal cords for all three proliferation markers, BrdU+ (after 2-h pulse), Ki67+ and pH3+ cells (Fig. 4h–l, arrows in Supplementary Fig. 7a–c). In agreement with our *ex vivo* analysis, an increase in the number of double BrdU+/pH3+ cells was also observed (Supplementary Fig. 7g,h), further suggesting faster cell-cycle progression. On the other hand, neuronal differentiation was severely impaired in *Nr5a2*-deleted embryos, as indicated by the expression of NeuN, DCX and βIII-Tubulin at protein level (Fig. 4n–q, larger magnifications in Supplementary Fig. 7d–f), as well as *NeuN*, *Dcx* and *Sox2* at mRNA level (Supplementary Fig. 5c–e). Similarly, NR5A2 deletion from the telencephalon of *E12.5* mouse embryos led to significant increase (Student's *t*-test) of proliferating progenitor cells (BrdU+ and pH3+ cells) and decrease in neurogenesis (βIII-Tubulin+ and NeuN+ cells), suggesting a general role for NR5A2 in early development of CNS (Fig. 5).

In addition, co-stainings of these embryos for proliferation and differentiation markers, further confirmed the notion that although proliferation is increased, neuronal differentiation is decreased in the same sections (Fig. 4q, larger magnifications in Supplementary Fig. 7f). Moreover, we showed that the ectopically proliferating cells in *Nr5a2 KO* embryos are not co-stained with markers for early-neuronal cells such as βIII-Tubulin (arrows in the rightmost panel in Supplementary Fig. 7f), suggesting that these cells remain NSCs, ectopically localized outside the VZ. Consistently, a strong increase in the numbers of apoptotic cells is evident in *Nr5a2 KO* and *Hetero* embryos (Supplementary Fig. 7i–k). These apoptotic cells were largely observed outside the VZ (Supplementary Fig. 7i,j,l), indicating that NSCs migrate outside the VZ, cannot differentiate into neurons and therefore initiate the programme for apoptosis in the hostile environment of MZ. Therefore, we suggest that the enhancement in apoptosis and decrease in neuronal production combinatorially cause the severe reduction in the size of CNS, despite the strong increase in the proliferation rates of NSCs. Furthermore, as a control experiment, under conditions of minimal dose of tamoxifen (∼0.05 mg per day as compared with ∼0.75 mg per day) none of these defects were observed (Supplementary Fig. 8).

We next examined the effect of *Nr5a2* deletion on astrogliogenesis. To this end, we showed that, as early as *E12.5*, a significant increase (Student's *t*-test) in early astrogliogenic markers is observed in *Hetero* and *Nr5A2 KO* spinal cords, including Sox9+, Aldh1l1+ and Nestin+ cells (Supplementary Fig. 9a–d). By real time RT–qPCR analysis, we also showed that the mRNA expression levels of *S100β*, *Gfap*, *Aldh1l1*, *Slc1a3* (encoding for Glast), *Fabp7* (encoding for Blbp) and *Cd44* are increased, although we cannot adequately detect some of them in the tissue by this early age (Supplementary Fig. 9e). To additionally test whether NR5A2 affects astrogliogenesis at later than *E12.5* developmental stages, we induced deletion of *Nr5a2* by applying tamoxifen to the pregnant mothers from *E12.5* to *E16.5* (Supplementary Fig. 9f–i). Similar to *E12.5*, analysis of these embryos at *E16.5* indicated higher numbers of Sox9+, Aldh1l1+, Nestin+, NFIA+ and GFAP+ cells in the spinal cord (Supplementary Fig. 9j–q), as well as increased mRNA levels for *S100β*, *Gfap*, *Nfia*, *Aldh1l1*, *Slc1a3*, *Fabp7*, *Cd44* and *Gja1* (encoding for Cx43) (Supplementary Fig. 9r). These observations suggest a repressive *in vivo* role for NR5A2 in astrogliogenesis, in good agreement with our *ex vivo* NSC data (Fig. 3u,v). Furthermore, neurogenesis was not affected in *E16.5* (Supplementary Fig. 10a–i), in good accordance with the observation that neurogenesis in mouse spinal cord is completed by *E14.5* (ref. 22). However, a mild effect on proliferation was evident (Supplementary Fig. 10j–q), likely reflecting the increase in astroglial progenitor cells, which are mostly scattered outside the VZ, all over the area of spinal cord (that is, BrdU+, Ki67+ and Sox2+ cells in Supplementary Fig. 10j–o).

Moreover, to specifically delete NR5A2 in NSCs, we crossed the *Nr5a2*^*fl/fl*^ with *Nestin-Cre* mice. However, we were not able to obtain viable offsprings out of these matings with the *Nr5a2*^*fl/+*^;*Nestin-Cre* genotype (*n=*5 crosses). From these crosses, an unusually high number of non-viable newly-born *P0* mice was observed. By genotyping newly-born, alive and non-viable embryos at *P0* (34 mice), we showed that all non-viable mice were carrying the *Nestin-Cre* allele (Supplementary Fig. 11a). Consistently, by analysing the CNS of *Nr5a2*^*fl/+*^;*Nestin-Cre* embryos, we showed that they suffer from severely malformed CNS and much reduced CNS size in *E14.5* (Supplementary Fig. 11b–c), reminiscent of our phenotype with the *Rosa26-CreER* inducible system. Collectively, NR5A2 is critically required for neural development and proper control of proliferation versus differentiation decisions of NSCs.

### NR5A2 induces neurogenesis via direct regulation of *Prox1*

**Figure 6:**
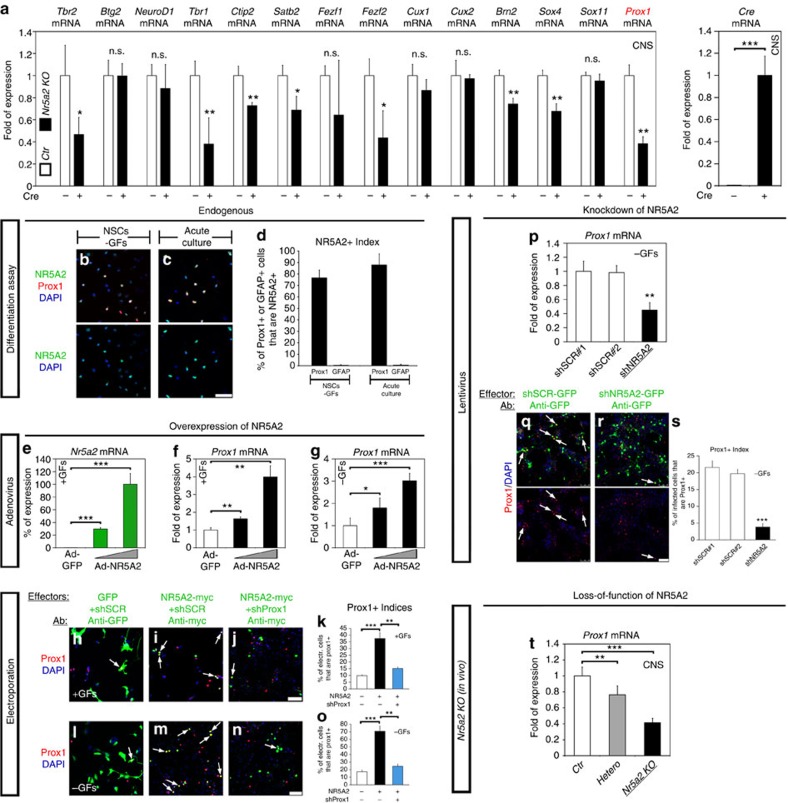
NR5A2 enhances the expression of *Prox1* gene. (**a**) RT–qPCR analysis in *E12.5* CNS of *Ctr* and *Nr5a2 KO* animals, as indicated. Values represent the mean±s.d. of four animals (*n=*4). (**b**,**c**) Double immunostainings of endogenous NR5A2 (green) with Prox1 (red) in differentiating NSCs (**b**) or acute cultures of *E14.5* spinal cord tissue (**c**). (**d**) Quantification of the Prox1+ or GFAP+ cells that express NR5A2 (% of marker+; NR5A2+/total marker+). (**e**–**g**) RT–qPCRs showing the quantifications of *Nr5a2* (**e**) and *Prox1* (**f**–**g**) mRNA levels in proliferating (**e**,**f**) or differentiating (**g**) NSCs, infected with Ad-GFP or Ad-NR5A2 viruses. (**h**–**j**, **l**–**n**) Double immunostainings of GFP or myc (green) with Prox1 (red) in NSCs co-electroporated (Amaxa) with GFP+shSCR (**h**,**l**), NR5A2-myc+shSCR (**i**,**m**) or NR5A2-myc+shProx1 (**j**,**n**) transgenes and cultured in the presence (**h**–**j**) or absence of GFs (**l**–**n**). Arrows indicate representative double positive cells. (**k**,**o**) Quantification of Prox1 index in +/− GFs, respectively. (**p**–**s**) RT–qPCR (**p**) and double GFP (green)/Prox1 (red) immunofluorescence analysis (**q**,**r**) indicating the downregulation of *Prox1* mRNA and protein levels, respectively, in differentiating NSCs infected with shNR5A2 compared with shSCR lentiviruses. Arrows indicate representative double GFP+/Prox1+ cells. Quantification of Prox1 index is indicated in **s** (% of GFP+; Prox1+/total GFP+). (**t**) RT–qPCR quantification of *Prox1* mRNA in *E12.5* CNS of *Ctr*, *Hetero* and *Nr5a2 KO* mice. The results are shown as mean±s.d. NS, not significant. *P*>0.05, *P<0.05, **P<0.01, ***P<0.001 (Student's *t*-test). Scale bars, 50 μm.

**Figure 7:**
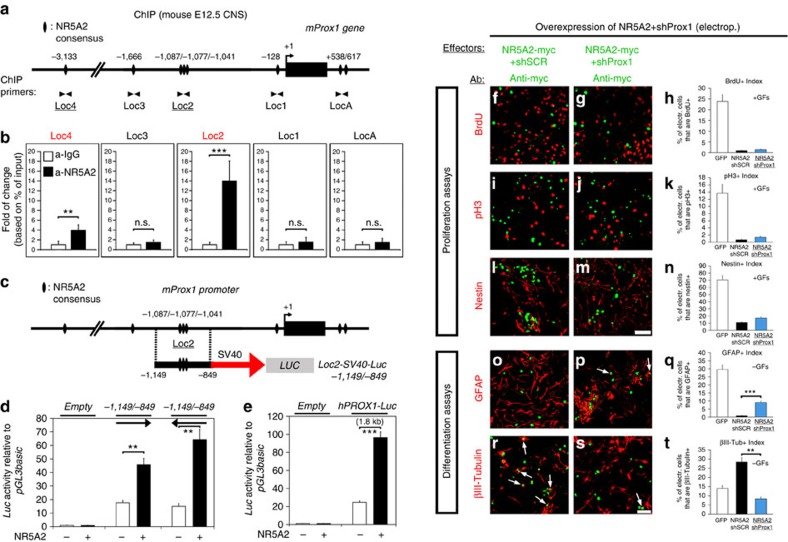
NR5A2 regulates Prox1 expression via direct activation of *Prox1* promoter. (**a**) Schematic representation of mouse *Prox1* gene locus. (**b**) ChIP analysis for the binding of NR5A2 to *Prox1* gene in chromatin prepared from the CNS of *E12.5* mouse embryos. (**c**) Schematic representation of the luciferase-reporter constructs containing the mouse DNA sequence of *Loc2* (−1,149/−849) in both orientations (*Loc2-SV40-Luc* (*−1,149/−849*)→/←). (**d**,**e**) Transcriptional assays in N2A neuroblastoma cells with mouse (**d**) or human (**e**) *Prox1* promoter constructs, as indicated. (**f**–**t**) Double immunostainings of myc (green) with various markers (red) in NSCs co-electroporated with NR5A2-myc+shSCR (**f**, **i**, **l**, **o** and **r**) or NR5A2-myc+shProx1 (**g**, **j**, **m**, **p** and **s**) expression vectors, respectively. Arrows indicate representative NR5A2-myc+ cells that co-express GFAP (**o**,**p**) or βIII-Tubulin (**r**,**s**). Quantifications of the indices of all markers are shown in **h**, **k**, **n**, **q** and **t** (% of GFP+ or NR5A2-myc+shSCR+ or NR5A2-myc+shProx1+; marker+/total GFP+ or NR5A2-myc+shSCR+ or NR5A2-myc+shProx1+). The results are shown as mean±s.d. NS, not significant. *P*>0.05, **P<0.01, ***P<0.001 (Student's *t*-test). Scale bars, 50 μm.

We next asked which is the mechanism(s) mediating the effects of NR5A2. Since neurogenesis was severely impaired on NR5A2 deletion in *E12.5* embryos, we screened *Nr5a2 KO* embryos for reduced mRNA levels of several genes encoding for transcription factors critical for neuronal differentiation. In agreement, we identified many such genes, including *Tbr2*, *Tbr1*, *Ctip2*, *Satb2*, *Fezf2*, *Brn2*, *Sox4* and *Prox1*, which were all downregulated (Fig. 6a). To further validate the downregulation of these genes, we normalized our RT–qPCR data against additional house-keeping genes, including *Ppia* (Supplementary Fig. 12a) and *Rpl13a* (Supplementary Fig. 12b). In all cases, we observed similar levels of downregulation. Moreover, the expression of a subset of genes encoding for transcription factors, not directly related to neuronal differentiation, were not affected, further confirming the downregulation of the neurogenesis-related genes (Supplementary Fig. 12c). In addition, by bioinformatic analysis, we were able to identify many consensus DNA-binding sites for NR5A2 in the promoters of all affected genes (Supplementary Fig. 13 and Fig. 7a). We and others have previously shown that Prox1 induces neurogenesis and suppresses proliferation and astrogliogenesis of NSCs^23^,^24^, suggesting that NR5A2 may directly promote Prox1 expression to mediate the same effects on NSCs.

To explore this scenario, we measured the percentage of NR5A2 and Prox1 double positive cells in NSCs and acute cultures of spinal cord. In both cases, the majority of Prox1+ cells were also NR5A2+ (Fig. 6b-d), while GFAP+ astrocytes were negative for both NR5A2 (Fig. 6d, arrowheads in Supplementary Fig. 1o,s) and Prox1 in the same cultures^23^. In agreement, overexpression of NR5A2 in NSCs induces Prox1 expression under proliferating and differentiating conditions at mRNA and protein levels (Fig. 6e–o). Conversely, shRNA or genetic ablation of NR5A2 expression are sufficient to significantly downregulate (Student's *t*-test) Prox1 expression in NSCs (Fig. 6p–s, Supplementary Fig. 14) or *Hetero* and *Nr5a2 KO E12.5* CNS (Fig. 6t). Next, by performing chromatin immunoprecipitation (ChIP) experiments in *E12.5* CNS, we showed that NR5A2 is sufficient to allow strong binding to a specific locus bearing three consecutive and conserved consensus DNA-binding sites for NR5A2 from −1,087 to −1,041 (*Loc2*). To a lesser extent, NR5A2 binds to an upstream locus −3,133 bp (*Loc4*) (Fig. 7a,b). To exclude the possibility of non-specific enrichment of these loci, we performed the same ChIP assays from chromatin prepared after Adeno-Cre infections of NSCs, isolated from spinal cords of *Nr5a2*^*fl/fl*^ mouse embryos (Supplementary Fig. 15a–c). These experiments showed that the enrichment of the previously identified loci was lost on Adeno-Cre-mediated *Nr5a2* gene deletion, further validating the *in vivo* ChIP assays. To further evaluate this interaction, we created two luciferase constructs containing the DNA sequence of *Loc2* (−1,149/−849) in both orientations, upstream of *SV40* promoter and luciferase-reporter gene (Fig. 7c). By performing transcriptional assays with these constructs in N2A neuroblastoma cells, we verified that NR5A2 strongly induces the activity of *Loc2*-*Prox1* promoter (Fig. 7d). Consistently, NR5A2 overexpression was sufficient to enhance the activity of human *PROX1* promoter (Fig. 7e). These data suggest that NR5A2 directly induces *Prox1* expression at the transcriptional level.

Next, to evaluate whether NR5A2 effects on NSCs are mediated by its regulatory action in *Prox1* promoter, we utilized a previously published shRNA vector targeting mouse *Prox1* (refs 25, 26) capable of blocking NR5A2-driven induction of Prox1 in NSCs (Fig. 6j,k,n,o). Thus, knockdown of Prox1 was not sufficient to rescue the effect of NR5A2 on NSC proliferation (BrdU+ and pH3+ indices) and identity (Nestin+ index) (Fig. 7f–n). On the contrary, it was sufficient to totally rescue the effect on neurogenesis (βIII-Tubulin+ index) and only partially the effect on astrogliogenesis (GFAP+ index) (Fig. 7o–t), suggesting that Prox1 is specifically required for NR5A2 effect on inducing neuronal differentiation. In agreement, overexpression of NR5A2 together with Prox1 resulted in much higher numbers of NSC-derived post-mitotic neurons than the overexpression of NR5A2 alone (Supplementary Fig. 16a–e), implying that this phenotype may be due to a functional and direct interaction between NR5A2 and Prox1 proteins. This interaction is very well-documented in other tissues and organs^27^,^28^,^29^,^30^. Immunoprecipitation and double immunofluorescence experiments confirmed this interaction in murine *E12.5* CNS and NSCs (Supplementary Fig. 16f–h). Moreover, considering that Prox1 promotes neurogenesis via negative regulation of Notch1 signalling^23^ (Supplementary Fig. 16i), we wanted to test whether NICD, the constitutively active form of Notch1, can recapitulate the effect of shProx1 on NR5A2 overexpression. Thus, similar to shProx1, NICD was able to fully rescue the effect of NR5A2 on neurogenesis, partially on astrogliogenesis and not at all on proliferation (Supplementary Fig. 16j–s). Collectively, these data suggest that NR5A2 promotes neuronal differentiation via direct binding and transcriptional activation of *Prox1* promoter and subsequent inhibition of Notch1 signalling.

### NR5A2 reduces proliferation via activation of *Ink4/Arf* locus

**Figure 8:**
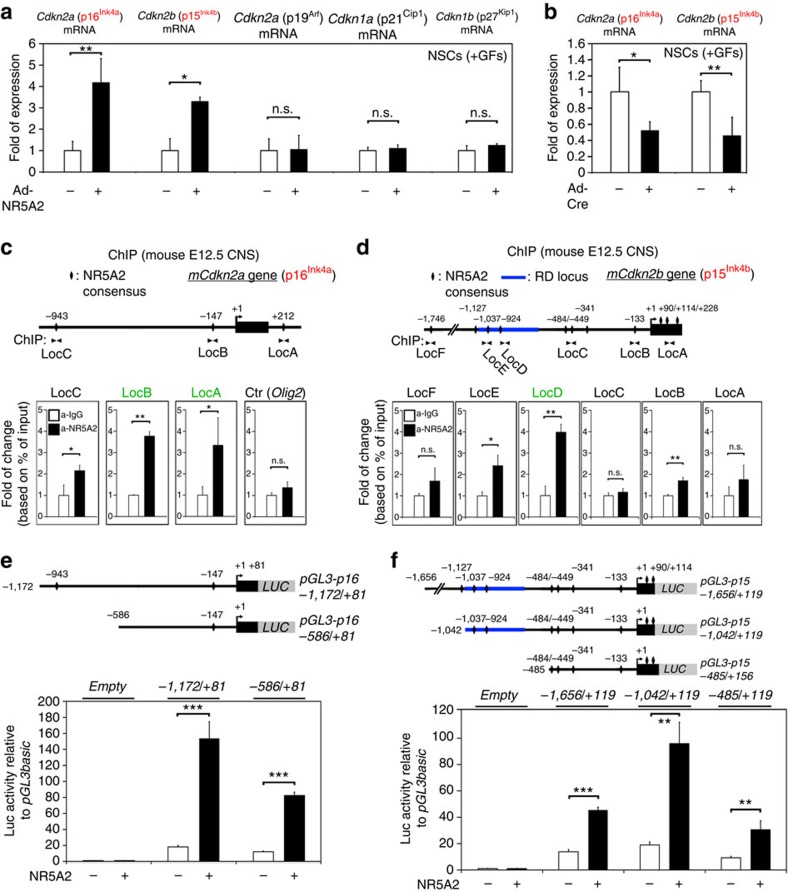
NR5A2 regulates the *Cdkn2a* (p16^Ink4a^) and *Cdkn2b* (p15^Ink4b^) genes of the *Ink4/Arf* genomic locus. (**a**) RT–qPCR quantification of various *Cdki* genes in NSCs infected with GFP- or NR5A2-expressing adenoviruses. (**b**) RT–qPCR quantification of *p16*^*Ink4a*^ and *p15*^*Ink4b*^ genes in *Nr5a2*^*fl/fl*^ NSCs derived from spinal cords and transduced with Ad-GFP or Ad-Cre (Supplementary Fig. 14). (**c**,**d**) Schematics of the organization of mouse *Cdkn2a* (**c**) and *Cdkn2b* (**d**) gene loci (upper panels). The lower panels present ChIP analyses for NR5A2 binding in chromatin prepared from *E12.5* CNS. (**e**,**f**) Schematic representation of a number of luciferase-reporter constructs containing various fragments of the mouse *p16*^*Ink4a*^ promoter (**e**) or *p15*^*Ink4b*^ promoter (**f**). The lower panels present transcriptional luciferase-reporter assays in N2A cells, as indicated. The results are shown as mean±s.d. NS, not significant. *P*>0.05, *P<0.05, **P<0.01, ***P<0.001 (Student's *t*-test).

However, the question of which molecular mechanism is responsible for the anti-proliferative action of NR5A2 in NSCs is still unanswered. To this end, we decided to test the effect of NR5A2 misexpression on cyclin-dependent kinase inhibitors (Cdkis), potent repressors of G1/S phase progression, including *Cdkn1a* (p21^Cip1^), *Cdkn1b* (p27^Kip1^), *Cdkn2a short-variant* (p16^Ink4a^), *Cdkn2a long-variant* (p19^Arf^) and *Cdkn2b* (p15^Ink4b^). Adenoviral-mediated overexpression of NR5A2 in NSCs specifically induces *p16*^*Ink4a*^ and *p15*^*Ink4b*^ genes of the *Ink4/Arf* locus (Fig. 8a). Conversely, Adeno-Cre-induced downregulation of *Nr5a2* gene expression in NSCs derived from *Nr5a2*^*fl/fl*^ mouse embryos caused a reduction in the expression of both *p16*^*Ink4a*^ and *p15*^*Ink4b*^ genes (Fig. 8b). By performing *in vivo* ChIP assays in *E12.5* mouse CNS, we showed that NR5A2 is directly recruited to specific consensus sites of the *p16*^*Ink4a*^ and *p15*^*Ink4b*^ promoters (Fig. 8c,d). NR5A2 extensively binds to *p16*^*Ink4a*^ promoter in two consensus sites, *LocA* and *LocB* (Fig. 8c) and to a lesser extent to *LocC*, while it is not detected in other unrelated loci, that is, *Olig2*. In *p15*^*Ink4b*^, NR5A2 is specifically recruited to *LocD* and to a lesser extent to *LocE* and *LocB* (Fig. 8d). Although statistical significant differences (Student's *t*-test) were observed for *LocC* of *p16*^*Ink4a*^ and *LocE* and *LocB* of *p15*^*Ink4b*^, we cannot totally exclude the possibility of non-specific binding of NR5A2 at these loci, due to low levels of enrichments as compared with control IgG. Interestingly, the region of strong NR5A2 binding (*LocD* of *p15*^*Ink4b*^) coincides with the polycomb-mediated repression domain of the *Ink4/Arf* locus (regulatory domain (RD); blue line in Fig. 8d). In addition, on Adeno-Cre-mediated deletion of *Nr5a2* gene expression in NSCs derived from *Nr5a2*^*fl/fl*^ mouse embryos, the NR5A2 recruitment in the *p16*^*Ink4a*^ and *p15*^*Ink4b*^ promoter loci was impaired (Supplementary Fig. 15d,e), further validating the *in vivo* ChIP assays. To further evaluate these interactions, we performed transcriptional assays with a set of *p16*^*Ink4a*^ and *p15*^*Ink4b*^ promoter luciferase constructs. In all cases, NR5A2 was sufficient to significantly induce (Student's *t*-test) the activities of these promoters (Fig. 8e,f), implying that NR5A2 promotes the expression of *p16*^*Ink4a*^ and *p15*^*Ink4b*^ genes to mediate its anti-proliferative action in NSCs. Notably, both Cdkis induce cell-cycle arrest through inactivation of E2F/phospho-pRb pathway^31^,^32^ and downstream suppression of cell-cycle promoting genes, *Cyclin E1*, *Cyclin A* and *Myc* (Supplementary Fig. 17a). In agreement, NR5A2 overexpression suppresses all three downstream targets of p16^Ink4a^ and p15^Ink4b^ pathway (Supplementary Fig. 17b–d).

### NR5A2 suppresses key components of JAK/STAT signalling

**Figure 9:**
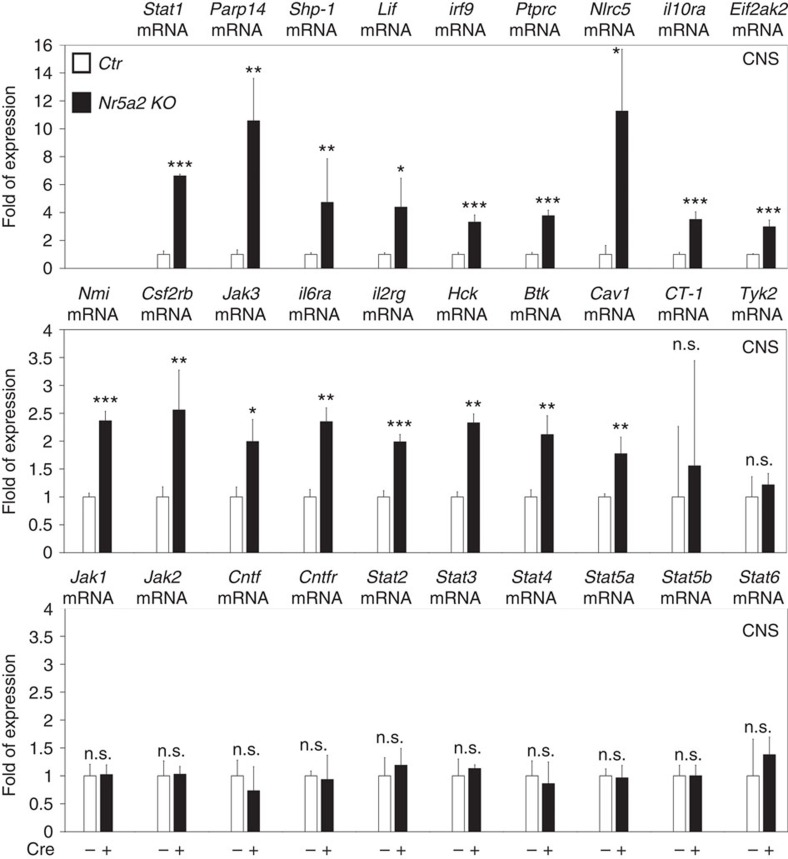
NR5A2 represses JAK/STAT signalling. RT–qPCR analysis in *E12.5* CNS of *Ctr* and *Nr5a2 KO* animals for the detection of mRNA levels of a number of genes involved in JAK/STAT pathway, as indicated. All values represent the mean±s.d. of four animals (*n=*4). NS, not significant. *P*>0.05, *P<0.05, **P<0.01, ***P<0.001 (Student's *t*-test).

Having shown that the anti-astrogliogenic effect of NR5A2 can only be partially rescued by shProx1 or NICD, we asked whether additional factors/pathways are involved in this function. Thus, we tested the involvement of the JAK/STAT. We focused on this pathway because, first JAK/STAT comprises the major positive regulatory pathway for the induction of astrogliogenesis^33^,^34^, and second NR5A2 is negatively regulated by this pathway (Fig. 2a,b,i–n). In agreement, the expression of 17 out of 29 tested genes of the JAK/STAT pathway was de-repressed in the CNS of *Nr5a2 KO E12.5* embryos, as compared with *Ctr* littermates (Fig. 9). The list of de-repressed genes includes *Stat1*, *Lif*, *Parp14*, *Shp-1*, *irf9*, *Ptprc*, *Nlrc5*, *il10ra*, *Eif2ak2*, *Nmi*, *Csf2rb*, *Jak3*, *il6ra*, *il2rg*, *Hck*, *Btk* and *Cav1*, indicating premature activation of JAK/STAT pathway in the CNS on deletion of NR5A2. Therefore, the suppressive function of NR5A2 on JAK/STAT pathway may also contribute to its ability to block astrogliogenesis.

## Discussion

**Figure 10:**
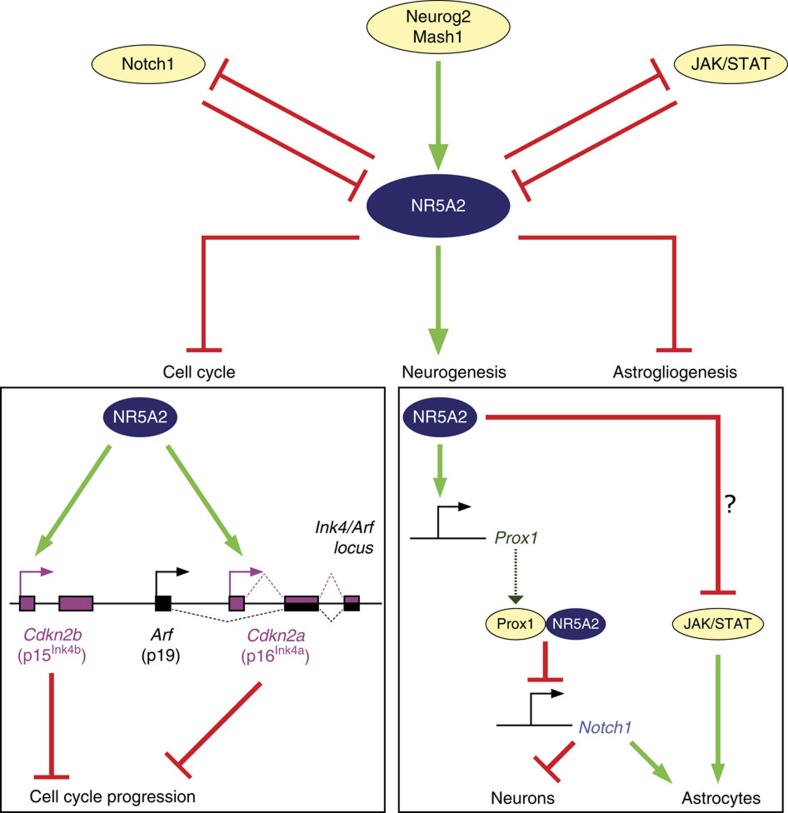
Schematic representation of our proposed model for the central role of NR5A2 in NSC fate specification. *NR5A2* is upstream regulated by the major neuronal and astrocytic pathways including proneural genes (*Neurog2* and *Mash1*), Notch1 and JAK/STAT signalling. On the other hand, NR5A2 regulates proliferation and differentiation of NSCs via direct regulatory effects on *Ink4*/*Arf* locus (*p16*^*Ink4a*^ and *p15*^*Ink4b*^), *Prox1* as well as Notch1 and JAK/STAT.

Based on the role of NR5A2 in ESCs^6^, iPSCs^9^,^10^, other progenitor and cancer cells^11^,^12^,^13^,^14^,^15^, we initially hypothesized that NR5A2 may promote proliferation of NSCs. Surprisingly, we showed that NR5A2 is a potent suppressor of NSC self-renewal and key player in neural fate decisions through direct regulatory effects on critical genes and pathways (Fig. 10—downstream). Upstream of NR5A2, a positive regulatory action is exerted by proneural genes and negative action by Notch1 and JAK/STAT, indicating negative feedback loops between NR5A2 and proliferative/astrogliogenic pathways (Fig. 10–upstream). Consistently, NR5A2 expression is associated with neuronal lineage and totally excluded from astrocytes during development (Fig. 1). Moreover, NR5A2 is expressed at low levels in actively proliferating NSCs of the VZ in early stages of spinal cord development (that is, *E10.5*; Fig. 1a,e–g), as compared with later stages (that is, *E12.5*; compare Fig. 1a with Fig. 1b). In these early stages, NR5A2 is probably repressed by the strong activity of Notch1 signalling and/or absence of sustained proneural gene expression (Fig. 2). At later neurogenic stages, activated proneural genes induce NR5A2 in the VZ progenitor cells of spinal cord to promote cell-cycle exit of NSCs and acquisition of neuronal identity (that is, *E12.5*; Fig. 1b,h-k). Then, at gliogenic stages of spinal cord development, NR5A2 is suppressed in progenitor cells by JAK/STAT and/or Notch1 to cease the neurogenic programme and allow initiation of astrogliogenesis (that is, *E16.5*; Fig. 1d,l–n).

In addition, here we show that NR5A2 promotes neurogenesis via direct activation of *Prox1* transcription (Figs 6 and 7). We also showed that NR5A2 and Prox1 form a protein complex in embryonic CNS. Therefore, we suggest that NR5A2 directly enhances the expression of its partner to repress downstream genes. This mode of NR5A2 action has been previously characterized for another co-repressor, namely SHP, which is also directly induced by NR5A2 (refs 29, 35, 36). For example, SHP interacts with NR5A2 to repress the expression of *Cyp7a1* in liver cells^35^,^36^,^37^ and *Cyp19a1* in adipocytes^38^. In our case, we propose that NR5A2 and Prox1 synergize to repress Notch1 and subsequently induce neurogenesis. Accordingly, we have previously reported that NR5A2 and Prox1 directly interact with the chromatin over the *Notch1* promoter to suppress *Notch1* activity^23^. Consistently, here we report that constitutively active Notch1 signalling rescues the effect of NR5A2 on inducing neuronal differentiation and faithfully phenocopies the action of Prox1 knockdown on this effect (compare Supplementary Fig. 16r,s with Fig. 7r–t).

To explain NR5A2 anti-proliferative effect, we focused on key inhibitors of G1/S phase progression. In particular, we exhibited the direct implication of NR5A2 in the activation of specific genes of the *Ink4/Arf* locus in NSCs, one of the most significant tumour suppressor loci in many organs, including brain cancers, glioblastomas and retinoblastomas^39^,^40^,^41^,^42^. More specifically, NR5A2 activates and interacts with chromatin over the promoters of *p16*^*Ink4a*^ and *p15*^*Ink4b*^ *in vivo* (Fig. 8). By exerting this direct regulatory action, NR5A2 arrests cell cycle, probably through inactivation of E2F/phospho-pRb pathway^31^,^32^. In good agreement, NR5A2 is sufficient to suppress the downstream effector genes of E2F/phospho-pRb pathway, namely *Cyclin E1*, *Cyclin A* and *Myc*. An alternative route for cell-cycle arrest is through *p19*^*Arf*^, which activates p53 and its downstream target *p21*^*Cip1*^ (refs 39, 43). However, NR5A2 is not able to induce *p19*^*Arf*^ or *p21*^*Cip1*^ (Fig. 8a). We therefore propose that NR5A2 is directly involved in the molecular mechanism that blocks proliferation of NSCs via the p15^Ink4b^–p16^Ink4a^–Cyclins–Myc axis in a p19^Arf^–p53–p21^Cip1^-independent manner. Moreover, it was previously shown that Bmi-1, a polycomb family transcriptional repressor, promotes propagation of NSCs by directly suppressing *p16*^*Ink4a*^ via RD domain^44^,^45^,^46^,^47^. Interestingly, we showed that NR5A2 directly interacts with the RD (Fig. 8d–f). These data suggest that NR5A2 may antagonize Bmi-1 over the RD to inhibit Bmi-1-mediated repression of *p16*^*Ink4a*^. Despite the positive regulatory action of NR5A2 in these tumour suppressor genes during nervous system development, it has been previously reported to act as an oncogene in other tissues^11^,^12^,^13^,^14^,^15^. Thus, here, we would like to propose a tumour suppressor function for NR5A2 specific for nervous system-related cancers via induction of *p16*^*Ink4a*^ and *p15*^*Ink4b*^ genes.

Finally, recent crystallographic analyses identified small molecules as potent ligands and pharmacological agonists/antagonists of NR5A2 (refs 2, 3, 4, 5). Accordingly, in a recent study it was shown that NR5A2 together with Retinoic Acid Receptor γ (Rarg) or their agonists facilitate the ability of neurogenic factors Mash1 (Ascl1), Brn2 and Neurog2 to convert mouse fibroblasts into functional and mature neurons^48^. These observations, together with our data in NSCs, could provide therapeutic possibilities for treatment of neurological disorders and inhibition of tumour progression in CNS.

## Methods

### Ethics statement

The study protocol was approved by the local ethics committee (Athens Prefecture Veterinarian Service; ϰ/3237/11-05-2012) and took place in the animal facilities of the Center for Experimental Surgery of the Biomedical Research Foundation of the Academy of Athens. All animals were kept in a 12-h light/12-h dark environment under constant temperature of 21 °C with free access to water and standard chow diet (TD.2016, Harland-Teklad Laboratories), grouped in cages of five individuals each and were handled in strict accordance with good animal practice as defined by the relevant US (Office of Laboratory Animal Welfare-NIH) and Greek animal welfare bodies.

### Generation of *in vivo* NR5A2 mutant models

To generate the temporal deletion of NR5A2 (tamoxifen-inducible *Nr5a2 KO* mice) in embryos, mice with homozygous floxed *Nr5a2* alleles (*Nr5a2*^*fl/fl*^) (kindly provided by Dr K. Schoonjans and Dr J. Auwerx, Laboratory of Integrative Systems and Physiology (LISP), École Polytechnique Fédérale de Lausanne (EPFL), Switzerland^11^,^12^,^21^) were crossed with a *Rosa26-CreERT* strain (no. 004847, Jackson Laboratories; kindly gifted from Dr A. Klinakis, BRFAA, Athens, Greece). This line (*Nr5a2*^*fl/+*^;*Rosa26-CreER)* was backcrossed and the pregnant *Nr5a2*^*fl/fl*^ mothers were treated with tamoxifen citrate (T9262, Sigma-Aldrich) via chow for 3 (*E9.5*–*E12.5*) or 4 (*E12.5*–*E16.5*) consecutive days (∼0.75 mg per day (∼0.2 mg per g feed) or ∼2 mg per day (∼0.55 mg per g feed), respectively, mixed in custom-made RM1 pellets) followed by i.p. administration of BrdU (2-h pulse for *E12.5* embryos, 3-h pulse for *E16.5* embryos) and sacrifice. The day of vaginal plug corresponds to *E0.5*. This resulted in the generation of mixed offsprings, containing embryos (*E12.5 or E16.5*) displaying 25% homozygous mutant (*Nr5a2*^*fl/fl*^*;CreER*–*Nr5a2 KO*), 25% heterozygous mutant (*Nr5a2*^*fl/+*^*;CreER*–*Hetero*) and 50% control (25% *Nr5a2*^*fl/fl*^, 25% *Nr5a2*^*fl/+*^*;without CreER*–*Ctr*) phenotypes. Total RNA and protein were extracted from CNS tissue of each genotype while embryos were separately embedded in paraffin blocks for sectioning. Deletion of *exon 5* of the floxed *Nr5a2* allele in CNS tissue was confirmed by RT–qPCR analysis with *mNr5a2-exon5*-specific primers.

To generate the *Nestin-Cre* conditional *Nr5a2 KO* mice (*Nr5a2*^*fl/+*^;*Nestin-Cre*), the *Nr5a2* floxed mice (*Nr5a2*^*fl/fl*^) were crossed with hemizygous *Nestin-Cre* transgenic mice. The *Nestin-Cre* mice, in which Cre-recombinase is expressed under the control of the *nestin* promoter in neural precursors^49^,^50^, were generated at Dr R. Kageyama lab (Kyoto University, Japan)^49^,^51^ and kindly provided to our laboratory by Dr F. Guillemot (MRC NIMR, London, UK). Viable and non-viable newly-born *P0* mice obtained from these crosses were genotyped by standard PCR using *cre*-specific primers. CNS specimens were dissected from *E14.5* mice and captured with a stereoscopic zoom microscope (Nikon SMZ800) equipped with a Retiga 2000 camera (Q Imaging).

Examination of embryonic and neonatal phenotypes was performed with mice of pure C57BL/6J background.

Genotyping and recombination events were determined by standard PCR using the following primers^21^:

ACE225: 5′- GTCATAGGGAGTCAGGATACCATGG -3′

ACE228: 5′- GTTCTGACCACTTTCATCTCCTCACG -3′

ACE231: 5′- GTTAGCAATTTGGCAGATTTACGC -3′

The *Cre* transgene was detected by primers^49^:

Cre-For: 5′- GCAAGAACCTGATGGACATGTTCAG -3′

Cre-Rev: 5′- GCAATTTCGGCTATACGTAACAGGG -3′or

Cre-For#2:5′- AAAATTTGCCTGCATTACCG -3′

Cre-For#2:5′- ATGTTTAGCTGGCCCAAATG -3′

To quantify the efficiency of recombination of the floxed *Nr5a2* locus in CNS tissue, we designed a real-time PCR assay (qPCR), based on ΔΔCt method for *exon5* normalized against *exon9* (Supplementary Fig. 5a). *Exon5* and *exon9* of *Nr5a2* were amplified using mNr5a2exon5-For/mNr5a2exon5-Rev and Nr5a2-avoid-For/Nr5a2-avoid-Rev primer pairs, respectively (for primer sequences see below and Supplementary Table 1, respectively). Quantification of recombination was calculated as % of non-recombined floxed *Nr5a2* locus.

### RNA extraction and real-time RT–qPCR analysis

Total RNA was isolated by TRI reagent solution (AM9738, Ambion|RNA, Life Technologies) according to manufacturer's instructions followed by treatment with RQ1 DNase (Promega, Madison, WI, USA). RNA concentration and purity were measured by Nanodrop 2000c (Thermo), and 1 μg was used for cDNA synthesis using the SuperScript First-Strand Synthesis System (Invitrogen, Carlsbad, USA) together with random hexamer primers. Quantitative Real time RT–PCR analysis was performed in a LightCycler 96 Instrument (Roche)^23^,^26^,^52^. Measured values were normalized using either *β-actin* or *Gapdh* mRNA levels as internal references. *Nr5a2* and *control* primer sequences used in RT–qPCR assays:

mNr5a2-For: 5′- TGAGTGGGCCAGGAGTAGTA -3′

mNr5a2-Rev: 5′- ATCAAGAGCTCACTCCAGCA -3′ (amplicon size: 90 bp)

mNr5a2exon5-For: 5′- AGCGAACTGTCCAAAACCAA -3′

mNr5a2exon5-Rev: 5′- TTCCAGCTTCATCCCAACAT -3′ (amplicon size: 131 bp)

mβactin-For: 5′- CCCAGGCATTGCTGACAGG -3′

mβactin-Rev: 5′- TGGAAGGTGGACAGTGAGGC -3′ (amplicon size: 141 bp)

mGapdh-For: 5′- CCAGTATGACTCCACTCACG -3′

mGapdh-Rev: 5′- CTCCTGGAAGATGGTGATGG -3′ (amplicon size: 97 bp)

The entire list of primer sets can be found in detail as Supplementary Table 1.

### Immunofluorescences

Regarding *in vivo* experiments, embryos or CNS specimens were fixed in 10% formalin solution for 24 h, dehydrated in alcohol, cleared in xylene and embedded in paraffin. Transverse sections (5-μm-thick) with respect to embryo body length axis were collected on poly-D-lysine-coated slides. For BrdU incorporation assays, *ex vivo* cultures were pulsed for 2 h with BrdU and then labelled with the appropriate anti-BrdU antibodies (mouse monoclonal (Dako, 1:300 dilution) or rat antibody (Abcam, 1:300 dilution)^52^). NR5A2 was detected using a rabbit polyclonal anti-NR5A2 antibody (kindly donated by Dr I. Talianidis, BSRC Alexander Fleming, Athens, Greece^53^,^54^,^55^,^56^, 1:1,000 dilution). Anti-GFP (1:1,500 dilution), anti-RFP (1:1,000 dilution) (detects DsRed) and anti-myc tag (1:100 dilution) were purchased from Molecular Probes (Invitrogen), Chromotek and Sigma, respectively. Prox1 was detected using either a rabbit polyclonal anti-Prox1 antibody (ReliaTech; 102-PA32, 1:200 dilution) or a mouse monoclonal antibody (Merck Millipore/Chemicon, 1:300 dilution)^23^,^26^. Mouse monoclonal antibodies against Nestin and Pax6 were obtained from Developmental Studies Hybridoma Bank (the University of Iowa, Iowa City, 1:100 dilutions) and mouse monoclonal anti-NeuN antibody was purchased from Merck Millipore/Chemicon (MAB377, 1:300 dilution). ca-Stat3 was detected using either a mouse monoclonal anti-Stat3 antibody (Atlas Antibodies; Product No. AMAb90776, 1:300 dilution) or a rabbit polyclonal antibody (Santa Cruz Biotechnology Inc.; C-20: sc-482, 1:300 dilution). Anti-GFAP monoclonal antibody was from Sigma (1:1,000 dilution), rabbit anti-active caspase 3 from Cell Signaling (#9661, 1:400 dilution), mouse monoclonal anti-βIII-tubulin from Covance USA (Tuj1, MMS-435P, 1:1,500 dilution), rabbit anti-pH3 from Abcam (1:500 dilution), mouse monoclonal anti-Ki67 from BD Pharmingen (Catalogue No. 550609, 1:100 dilution) and rabbit anti-CyclinD1/-CyclinE1 from Santa Cruz Biotechnology Inc (1:100 dilutions). DCX and Mash1 were detected using goat anti-DCX and mouse anti-Mash1 antibodies (1:100 dilutions), respectively, and were kindly provided by Dr F. Guillemot. Secondary antibodies conjugated with AlexaFluor 488 (green), 568 (red) or 647 (far red) were purchased from Molecular Probes (1:800 dilutions). All antibodies were detected with standard immunofluorescence experimental protocol^23^,^25^,^26^,^52^. Cell nuclei were labelled with DAPI (1:2,000 dilution, Molecular Probes). Specimens were viewed and analysed with confocal microscopy (Leica TCS SP5 on a DMI6000 inverted microscope). Statistical analysis was performed with the two-tailed paired Student's *t*-test.

### Western blot analysis

Total protein extracts from *Ctr* and *Nr5a2 KO* mouse embryos were obtained with TRI reagent solution following RNA extraction according to manufacturer's protocol, until the protein resuspension step^57^,^58^. Particularly, proteins were isolated from the phenol-ethanol supernatant layer left over after the DNA precipitation step by isopropanol precipitation. Protein pellets were resuspended in 1% SDS, incubated 20 min in a 50 °C water bath and completely dispersed by sonication (VCX, Sonics & Materials Inc.: 60% amplitude, 3–5 cycles of 15 s with 30 s ice incubation) (modified protocol). Samples were then clarified by centrifuging at 10,000*g* at 4 °C to sediment insoluble material. The clear supernatants containing the total solubilized proteins were subjected to immunoblot analysis with a rabbit custom-made polyclonal antibody (1:1,000 dilution) raised against the mouse peptide of NR5A2 (produced and purchased from Davids Biotechnologie GmbH-Custom Antibodies, Regensburg, Germany; amino acid sequence: HSASKGLPLSHVALPPTDYDR; HPLC-purified peptide). Protein electrophoresis, transfer and western blotting were performed with the standard protocol^25^. Protein loads were verified with β-actin as reference protein, using a primary mouse monoclonal anti-β-actin antibody (Sigma, 1:10,000 dilution).

To test the specificity of anti-NR5A2 antibodies, immunoblot analyses were performed in NSCs or N2A cells that were electroporated (Amaxa) or lipofected (Lipofectamine 2000, Invitrogen), respectively, with NR5A2-myc (+) or pcDNA3 (−) expression vectors. Both the custom-made antibody (1:2,000 dilution) and the antibody kindly donated by Dr I. Talianidis (1:3,000 dilution) were used to obtain signal from the NR5A2 protein (Supplementary Fig. 18).

To perform the peptide competition assays (Supplementary Fig. 18), pre-incubation of the custom-made antibody with immunogen peptide (blocking peptide) was done for 2 h at RT in blocking solution-free buffer, with mixing. At the end of pre-incubation, blocking solution was added and the mixture was poured onto the membrane (or onto cells when performing immunofluorescence experiments) for overnight incubation at 4 °C. Preabsorption of the antiserum with the corresponding immunogen peptide totally competed out the western blot bands (or the immunostaining). Immunogen peptide was not available for the antibody kindly donated by Dr I. Talianidis.

All uncropped western blots can be found in Supplementary Fig. 19.

### Histological analysis (H&E stainings)

*E12.5* embryos were fixed in 10% buffered formalin, embedded in paraffin and sectioned transversely at 5 μm. Progressive haematoxylin and eosin (H&E) routine stain was used for morphometrical purposes (Haematoxylin: Fluka, AG, Switzerland; eosin Y: alcohol and water soluble, Winlap, UK) in spinal cord and telencephalon specimens. Poly-D-lysine-coated slides were mounted in fluorescence-free DPX mountant (Sigma-Aldrich) and examined under digital imaging microscope (inverted Leica DFC-500) equipped with digital cooled CCD camera for high-resolution bright field imaging.

### Chromatin immunoprecipitations

To analyse the molecular interactions of NR5A2 in nervous system, ChIP experiments were carried out in: (i) cells derived from total CNS of *E12.5* mouse embryos (*in vivo*; Fig. 7a,b and Fig. 8c,d) or (ii) NSCs derived from *E14.5* spinal cords of *Nr5a2*^*fl/fl*^ mice (*Nr5a2*^*fl/fl*^ *ex vivo* NSCs) that were transduced three times with Ad-GFP or Ad-Cre (see ‘culture of NSCs, overexpression and knockdown studies' and Supplementary Fig. 15)^23^,^25^,^26^. To cross-link proteins to chromatin, cells were treated with 1% formaldehyde for 10 min at room temperature and cross-linking was stopped by adding glycine to a final concentration of 0.125 M. Following dounce homogenization, cell nuclei were obtained and lysed with sonication in 1% SDS-containing buffer to an average length of 150–500 bp (VCX: 30% amplitude, 1 s intervals, 16-min total time, tube on ice-ethanol (−16° C)). After soluble chromatin was pre-cleared with agarose beads, BSA and t-RNA, 50 μg of sheared DNA, extracted from CNS of *E12.5* mouse embryos, were used per IP reaction with 10 μg of antibody. For ChIPs carried out in *Nr5a2*^*fl/fl*^ *ex vivo* NSCs, 50 μg of DNA with 5 μg of antibody were used. Chromatin–antibody immunocomplexes were formed overnight at 4 °C using affinity-purified antibody to NR5A2 (kindly donated by Dr I. Talianidis) or control IgG antibody. The antibody bound chromatin was retained on protein-A magnetic beads (Invitrogen, Dynal). After 6 h of reverse-cross-linking at 65 °C, and digestion with RNAse A (10 μg ml^−1^) and proteinase K (20 μg ml^−1^), DNA was purified from the immobilized bound immunocomplexes using the Qiagen PCR purification minelute kit. Detection and analysis of ChIP precipitates were performed by qPCR. Assay repeatability was determined using quadruplicate reactions. Real-time PCR was carried out using the Platinum SYBR Green qPCR supermix-UDG kit (Invitrogen #11733–046) in a 25 μl qPCR reaction following the manufacturer's specifications. In all cases, data (*C*_t_ values) derived from the input sample were used for normalization by the 'per cent of Input (% IP)' method and presented as fold of change relative to control anti-IgG IPs. All ChIP assays were repeated at least four times.

The sequence of the core consensus response element for NR5A2 (NR5A2RE) was identified on each promoter sequence based on the previously published and well-characterized motif CAAGG^8^,^59^,^60^, found on the plus and minus strands in both orientations. The following pairs of primers were used to amplify the genomic loci from mouse *Prox1, Cdkn2a* (p16^Ink4a^) and *Cdkn2b* (p15^Ink4b^) genes, as indicated in Figs 7a and 8c,d, respectively:

#### Prox1 gene

LocA-For: 5′- GTATCTTCACCCGGTTGCTG -3′

LocA-Rev: 5′- CGATTCATGTAAATAACACC -3′

Loc1-For: 5′- GTTCTCTTGCCTCGCTATCC -3′

Loc1-Rev: 5′- CTCCGCTCCACAACAAGATT -3′

Loc2-For: 5′- CTGTTAACTGTGCCCAGGGAGAGGA -3′

Loc2-Rev: 5′- TGGTTTGACATCTTGGGTGA-3′

Loc3-For: 5′- CCCGACTTGAGCTCGGGAAAGTCTT -3′

Loc3-Rev: 5′- AGAGAGGTAGGTGGGTGTGC-3′

Loc4-For: 5′- GCTGCCACAGCTGCTACTTG-3′

Loc4-Rev: 5′- TGCTGCACGTCGCCTAGAGG-3′

#### *Cdkn2a* (p16^Ink4a^) gene

LocA-For: 5′- TCGTACCCCGATTCAGGTAG-3′

LocA-Rev: 5′- ATCTGGGGTATGCATTTCAA-3′

LocB-For: 5′- CCTGAACCCTGCATCTCTTC-3′

LocB-Rev: 5′- GCCATAGGTGGCGCTATTT-3′

LocC-For: 5′- AATGCCAGGCCTTTAATCCT-3′

LocC-Rev: 5′- GTCCTCACCAGAAAGGCAAT-3′

CTR (*Olig2*)-For: 5′- CCCTCCTGTTGTCTCTCCTG-3′

CTR (*Olig2*)-Rev: 5′- TTGGGATTATTCCATTCCACA-3′

#### *Cdkn2b* (p15^Ink4b^) gene

LocA-For: 5′- TCACCGAAGCTACTGGGTCT-3′

LocA-Rev: 5′- CTGTGGCAGAAATGGTCCTT-3′

LocB-For: 5′- TGAGTGCAGAGGCACAACTT-3′

LocB-Rev: 5′- GGGGTGGTATCTGAGAGTCG-3′

LocC-For: 5′- ACCAAAGCTCAAGGGGAAAA-3′

LocC-Rev: 5′- GGCGGTATTTCACAGTTAGCA-3′

LocD-For: 5′- GGTCTCCCCTAGCAGGATTC-3′

LocD-Rev: 5′- GCCTGTCATTAAACAGGGTGA-3′

LocE-For: 5′- GCACCTGGCTTCCTTTAAGA-3′

LocE-Rev: 5′- CTGTTGCCAAACAACTCTGG-3′

LocF-For: 5′- GGAAGCGGAGAGCAGAGATA-3′

LocF-Rev: 5′- GTGCCACATTCTCCCACTTT -3′

### Co-immunoprecipitation assays

Co-immunoprecipitation assays were performed from embryonic mouse CNS tissue (*E12.5*), including both brain and spinal cord^23^. Briefly, tissues were lysed in 50 mM Tris HCl (pH 7.4), 150 mM NaCl, 1% Nonidet-P-40 (NP-40), 0.1% deoxycholate (DOC), 0.025% SDS, 1 mM EDTA, 1 mM phenylmethylsulfonyl fluoride (PMSF) and cocktail protease inhibitors (Sigma). The following antibodies were used: anti-NR5A2 (kindly donated by Dr I. Talianidis, 5 μg of antibody per IP reaction), anti-Prox1 (1:500 dilution for western blot analysis) and control IgGs from Dako. To retain antibodies protein–A agarose beads were utilized (Pierce).

### Luciferase assays

Luciferase-reporter assays were performed with luciferase/β-galactosidase kits (Promega)^23^,^25^,^26^,^52^. In detail, N2A cells (mouse neuroblastoma cell line) were plated in 12-well plates at a density of 2 × 10^5^ and were maintained overnight in DMEM supplemented with 10% (*vol/vol*) heat-inactivated FBS and pen-strep (100 U ml^−1^ and 100 μg ml^−1^, respectively; Invitrogen). The next day, N2A cells were transiently transfected with Lipofectamine 2000 (2 μl per well) with 0.75 μg of each reporter construct, 1.5 μg of NR5A2 (+) or GFP (−) expression vectors and 0.15 μg of a β-galactosidase expression plasmid to normalize for transfection efficiency. The transfection mixture was replaced 6 h later by growth medium and the cells were further cultured for 42 h. About 48 h after transfection, cells were lysed and luciferase activity was determined according to manufacturer's specifications (Promega) with a Lumat LB 9507 luminometer (Berthold Technologies). To correct for differences in transfection efficiencies, luciferase activity was normalized to that of β-galactosidase, which was measured spectrophotometrically at 420 nm using *o*-nitrophenyl β-D-galactopyranoside as substrate. All luciferase experiments were repeated at least four times and data are represented as the mean±s.d. of quadruplicate assays.

In this study, we used the following Luciferase-reporter constructs for the transcriptional assays: *Prox1* *promoter*—both orientations of mouse *Loc2* sequence (including three consensus binding sites for NR5A2) (−1,149/−849) upstream of the *SV40* promoter and luciferase (*Loc2-SV40-Luc (−1,149/−849)*→/←) were constructed for this study by PCR with genomic DNA isolated from total brain of *E12.5* mouse CNS and standard cloning methodology, 1.8 kb of human *Prox1* promoter fused with luciferase (*hProx1-Luc*-*1.8* kb; kindly gifted from Dr Y.K. Hong^61^) and empty vector (*pGL3 basic*).

#### *p16* ^*Ink4a*^ promoter (mouse)

1,172 and −586/+81 fragments from the transcription start site (TSS) (*pGL3-p16 (−1,172/+81)* and *pGL3-p16 (−586/+81)*), kindly gifted by Dr H. Nakauchi and Dr A. Kamiya^27^.

#### *p15* ^*Ink4b*^ promoter (mouse)

1,656, −1,042/+119 and −485/+156 fragments from the TSS (*pGL3-p15 (−1,656/+119)*, *pGL3-p15 (−1,042/+119)*; kindly gifted from Dr J. Bies and Dr L. Wolff^62^ and *pGL3-p15 (−485/+156)*; kindly gifted from Dr A. Pellicer^63^).

### Culture of NSCs and gain- and loss-of-function studies

NSC primary cultures of embryonic (*E14.5*) mouse spinal cords were prepared and maintained as neurospheres^23^,^26^,^52^. Proliferation or differentiation studies were performed after dissociation of NSCs to single cells, plating onto poly-L-lysine (Sigma) coated coverslips in 24-well plates at a density of 1.5 × 10^5^ and further *ex vivo* culture for 2 or 3 days with or without GFs, respectively, in a 37 °C humidified incubator with 5% CO_2_ (Supplementary Fig. 2a). Detection of NSCs undergoing apoptosis on single-cell level was carried out with active caspase 3 staining.

For NR5A2-myc (*pcDNA3*), NR5A2-YFP (*pEYFP*) (kindly donated by Dr I. Talianidis), Prox1 (*pcDNA3*), Neurog2-DsRed (*pCAG-IRES*; a generous gift from Dr B. Berninger, Johannes Gutenberg University Mainz, Germany), Mash1 (*pGaggs*), NICD-myc (*pcDNA3.1*; kindly provided by Dr T. Kadesch, the University of Pennsylvania, Philadelphia, PA), ca-Stat3 (*pMXs*; obtained from Dr S. Yamanaka, Kyoto University, Japan) or control GFP (*pcDNA3* or *pmax*) overexpression, NSCs were transfected using an Amaxa electroporator (Lonza) with 6 μg of plasmid DNA per electroporation, according to manufacturer's instructions^23^,^26^.

Construction of the recombinant control (Ad-GFP) and NR5A2 (Ad-NR5A2-GFP #1, #2)-overexpressing adenoviruses was performed using the pAd/PL-DEST Gateway vector (ViraPower Adenoviral Expression System, Invitrogen Life Technologies), according to manufacturer's instructions. Briefly, the cDNAs encoding wild-type *Nr5a2* and *eGFP* were cloned into a modified version of the *pENTR.GD* entry vector and introduced into the *Destination* vector (kindly provided by Dr M. Xilouri and Dr A. Klinakis, BRFAA, Athens, Greece). Recombinant adenoviral particles were produced by homologous recombination into the HEK-293A cell line, with virus titres determined by the plaque assay method^64^. The generated adenoviral supernatants were then used to infect primary NSCs for 6 h at a multiplicity of infection (MOI) of 10. The exogenous expression of *Nr5a2* was confirmed by RT–qPCR (Fig. 6e). In addition, NSCs derived from *E14.5* spinal cords of either *Nr5a2*^*fl/fl*^ mice (*Nr5a2*^*fl/fl*^ NSCs) or *Rosa26-(td)Tomato* mice (kindly provided from Dr L. Zagoraiou (BRFAA, Athens, Greece); *Rosa26-(td)Tomato* NSCs) were transduced three times with Cre-recombinase–expressing adenovirus (Ad-Cre-GFP), generously gifted by Dr A. Klinakis and Dr T. Rampias^65^. Genomic DNA was isolated using TRI reagent solution according to manufacturer's protocol. Following RNA extraction, DNA was precipitated from the interphase/organic layer with ethanol and used for genotyping PCR.

For shRNA knockdown studies, lentiviral vectors with the phosphoglycerate kinase (*hPGK*) promoter driving *eGFP* expression were produced in HEK-293T cells as described and recommended by the supplier (Sigma, TRC lentiviral Library). Viral transductions with lentiviruses expressing shSCRs, shNR5A2 or shProx1 (against mouse sequences) were performed overnight at a MOI of 10 in: (i) NSCs or: (ii) neuronal progenitors isolated from *E16.5* mouse spinal cords and cultured *ex vivo* (Supplementary Fig. 4a–e) in serum-free medium containing Neurobasal (Gibco, cat. #21103-049), 2% B-27 supplement (Gibco, cat. #17504-044), 250 μM L-glutamine (Sigma, cat. #G7513) and 50 μg per ml gentamicin antibiotic (or pen-strep) (Sigma). The reference number and corresponding sequence for each shRNA construct used in this study can be found in Sigma's TRC library webpage or can be provided on request to the authors of this manuscript. The shProx1 lentiviral construct was previously identified and described^25^,^26^.

Each set of electroporation or viral transduction was performed in four independent experiments.

### Statistical analysis

Before analysis, the normal distribution of values was verified with the Shapiro–Wilk normality test using IBM SPSS Statistics for Windows, Version 20.0; in all cases the probabilities were >0.05, denoting normally distributed data sets. For statistical analysis, the measurements and experimental values from independent experiments were estimated with the two-tailed paired Student's *t*-test. All the results are shown as mean±s.d. values of at least four independent experiments. The exact *P* values are described and specified in each figure legend. *P* values <0.05 (*P*<0.05) were considered statistically significant. All analyses were done using Microsoft Excel 2013 or GraphPad Prism 6.05 softwares.

### Data availability

The authors declare that all data supporting the findings of this study are available within the article and its Supplementary Information files. All data presented in this study are available from the authors upon request.

## Additional information

**How to cite this article:** Stergiopoulos, A. *et al*. Nuclear receptor NR5A2 controls neural stem cell fate decisions during development. *Nat. Commun.* 7:12230 doi: 10.1038/ncomms12230 (2016).

## Supplementary Material

Supplementary Information Supplementary Figures 1-19, Supplementary Table 1 and Supplementary References
