# Supplementary material for: Nuclear receptor NR5A2 controls neural stem cell fate decisions during development
Source: Nat Commun. 2016 Jul 22;7:12230. doi: 10.1038/ncomms12230 (PMC4961839; doi:10.1038/ncomms12230)
Supplement: Supplementary Information — Supplementary Figures 1-19, Supplementary Table 1 and Supplementary References [file ncomms12230-s1.pdf]

Supplementary Figure 1

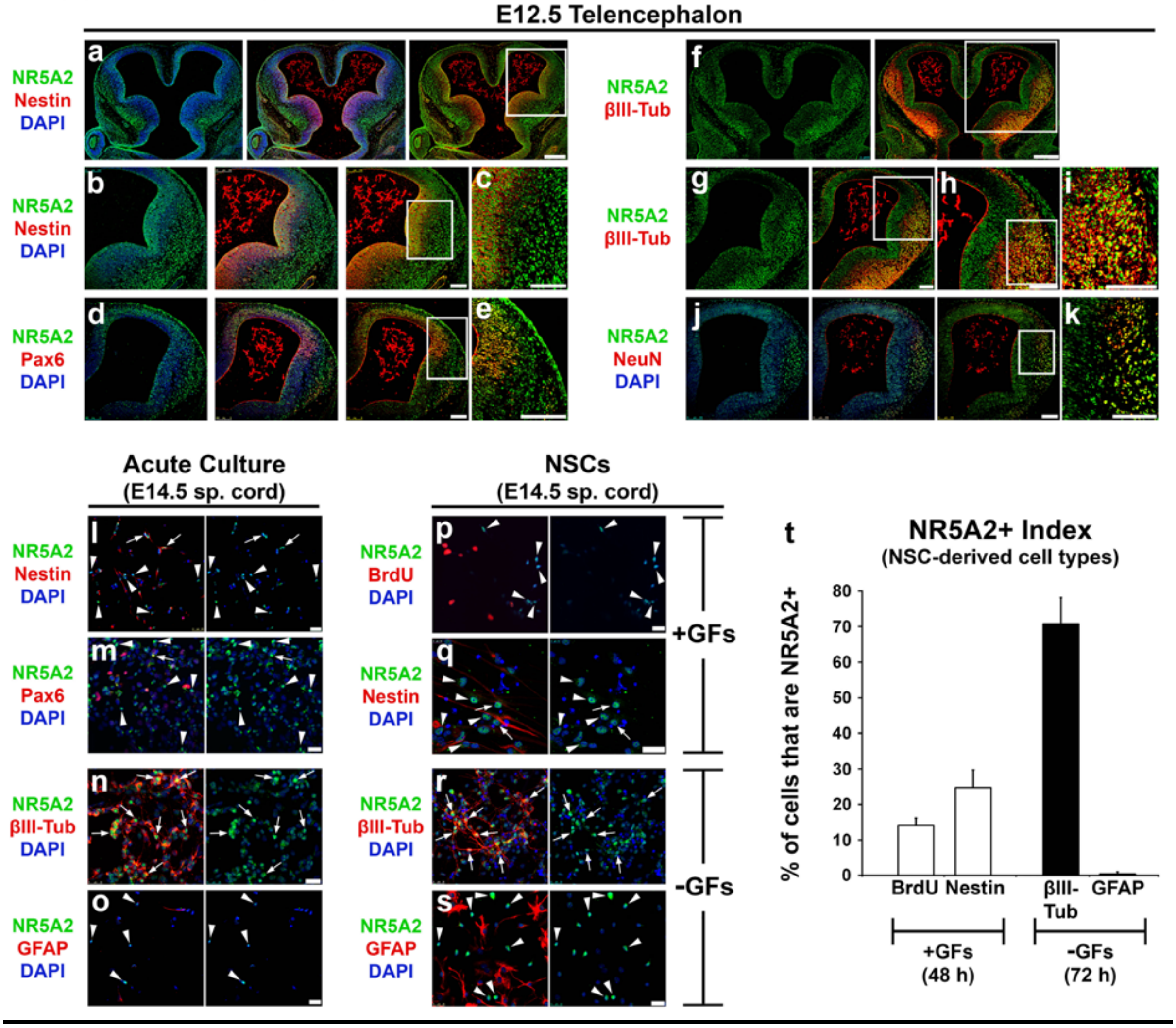

Supplementary Figure 1. NR5A2 expression pattern in *E12.5* mouse telencephalon and *ex vivo* cultured spinal cord cells.

(**a-k**) Double immunostainings of NR5A2 (green) with Nestin (a-c), Pax6 (d-e), βIII-Tubulin (f-i) or NeuN (j-k) (all red) at *E12.5* mouse telencephalon, as indicated. (b), (c), (e), (g), (h), (i) and (k) micrographs are larger magnifications of the square shapes depicted in (a), (b), (d), (f), (g), (h) and (j), respectively. Control immunofluorescences without primary antibody showed no staining.

(**l-s**) Double immunostainings of NR5A2 (green) with Nestin (l, q), Pax6 (m), BrdU (p), βIII-Tubulin (n, r) or GFAP (o, s) (all red) in *acute* cultures of spinal cord tissue (l-o) and *E14.5* NSCs (p-s) cultured *ex vivo* in the presence (+GFs) (p-q) or absence of growth factors (-GFs)

(r-s). (p) NSCs were pulsed for 2 h with BrdU and then labeled with the appropriate anti-BrdU primary antibody. Representative NR5A2+ cells are indicated with arrows while NR5A2- cells with arrowheads.

(**t**) Quantification of the cell populations that express NR5A2 (% of marker+; NR5A2+ / total marker+). The results are shown as mean ±SD.

Cell nuclei were visualized with DAPI staining (blue). Scale bars: (a, f) 250 μm; (b-e, g-k) 100 μm; (l-s) 25 μm.

Supplementary Figure 2

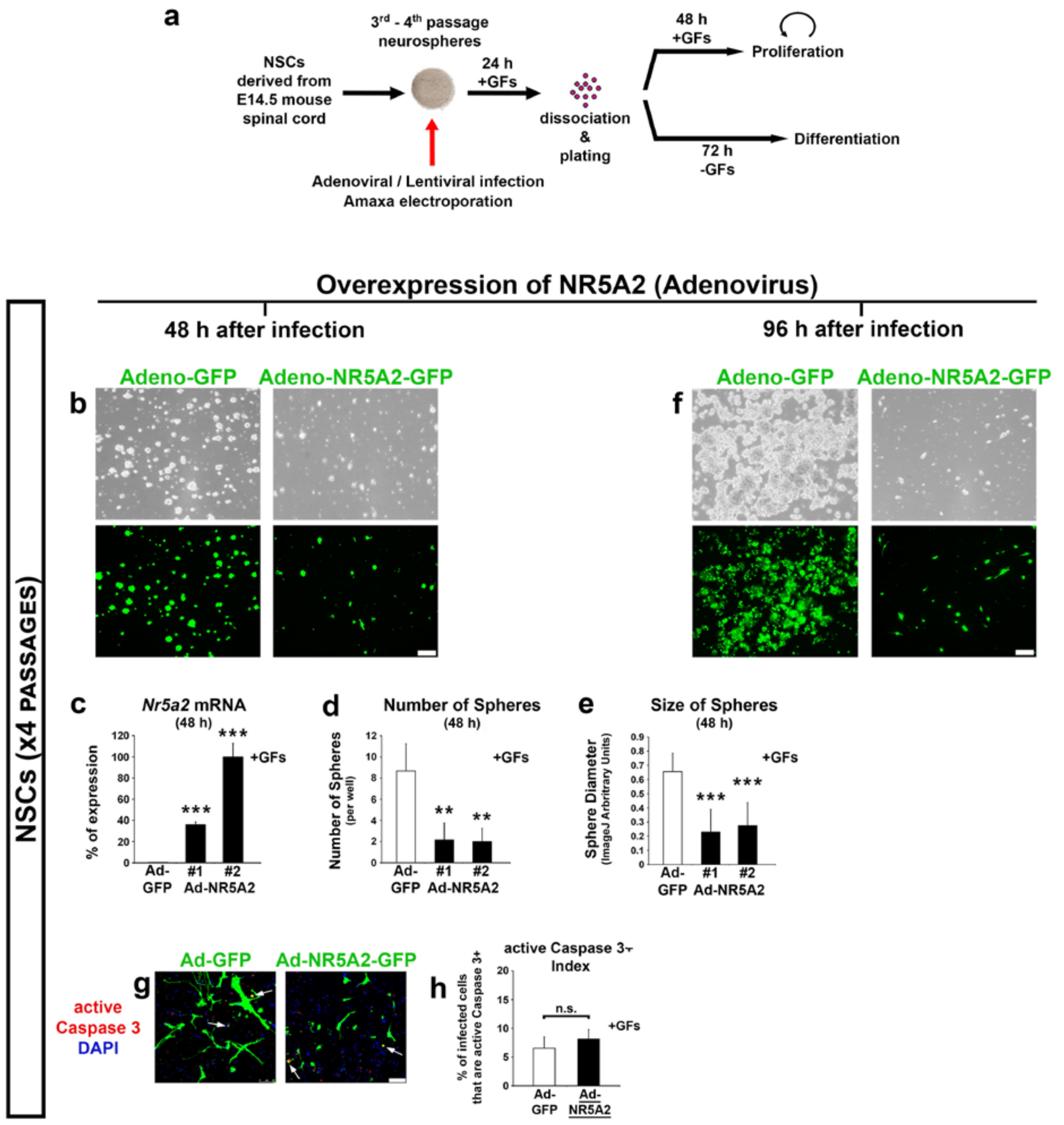

<u>Supplementary Figure 2</u>. Overexpression analysis of NR5A2 in spinal cord-derived NSCs.

(**a**) Schematic representation of the protocols used for Adenoviral/Lentiviral infections and Amaxa electroporation of NSCs derived from embryonic (*E14.5)* mouse spinal cord and cultured *ex vivo*. Neurosphere cultures were passaged at least three times before infection or electroporation. Neurospheres were cultured for another 24 h and then dissociated and plated

in the presence or absence of GFs for 48 or 72 h, in order to measure proliferation and differentiation indices, respectively.

(**b and f**) Phase contrast (upper panels) and fluorescent images (lower panels) of NSCs infected with GFP (Adeno-GFP) or NR5A2-GFP (Adeno-NR5A2-GFP#1, #2) adenoviruses, 48 h (b) or 96 h (f) after infection.

(**c**) Quantitative real-time RT-PCR analysis of the infected NSCs.

(**d-e**) Quantification of the number (d) and size (e) of neurospheres 48 h post-infection.

(**g-h**) Double GFP (green)/active Caspase 3 (red) immunostainings of NSCs transduced with Ad-GFP or Ad-NR5A2-GFP adenoviruses (g) and quantification of apoptosis (h) (% of GFP+; active Caspase 3+ / total GFP+).

In each case, arrows indicate GFP+ cells that are co-localized with each marker. The results are shown as mean ±SD. n.s.: not significant P>0.05, ** P<0.01, *** P<0.001 (Student's *t*-test). Scale bars: (b, f) 100 μm; (g) 50 μm.

Supplementary Figure 3

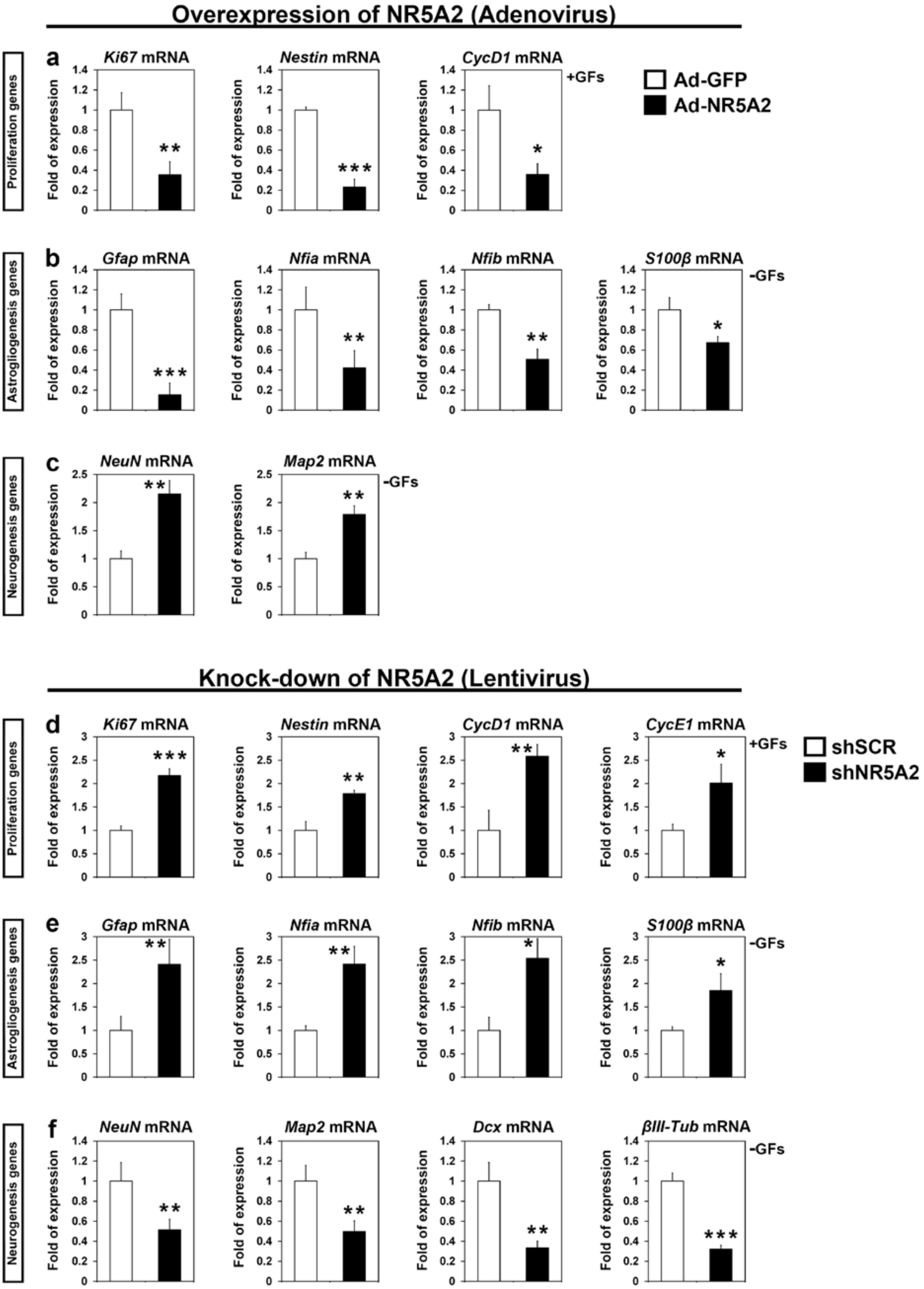

<u>Supplementary Figure 3</u>. RT-qPCR analyses in proliferating or differentiating NSCs

infected with adeno (overexpression studies)- or lenti (knock-down studies)- viruses.

(**a-c**) Quantifications of mRNA levels of genes related to proliferation (+GFs) (a), astrogliogenesis (-GFs) (b) or neurogenesis (-GFs) (c) in NSCs infected with Ad-GFP or Ad-NR5A2 viruses.

(**d-f**) Quantifications of mRNA levels of genes related to proliferation (+GFs) (d), astrogliogenesis (-GFs) (e) or neurogenesis (-GFs) (f) in NSCs infected with lentiviruses expressing shNR5A2 compared to shSCR.

The results are shown as mean ±SD. * P<0.05, ** P<0.01, *** P<0.001 (Student's *t*-test).

Supplementary Figure 4

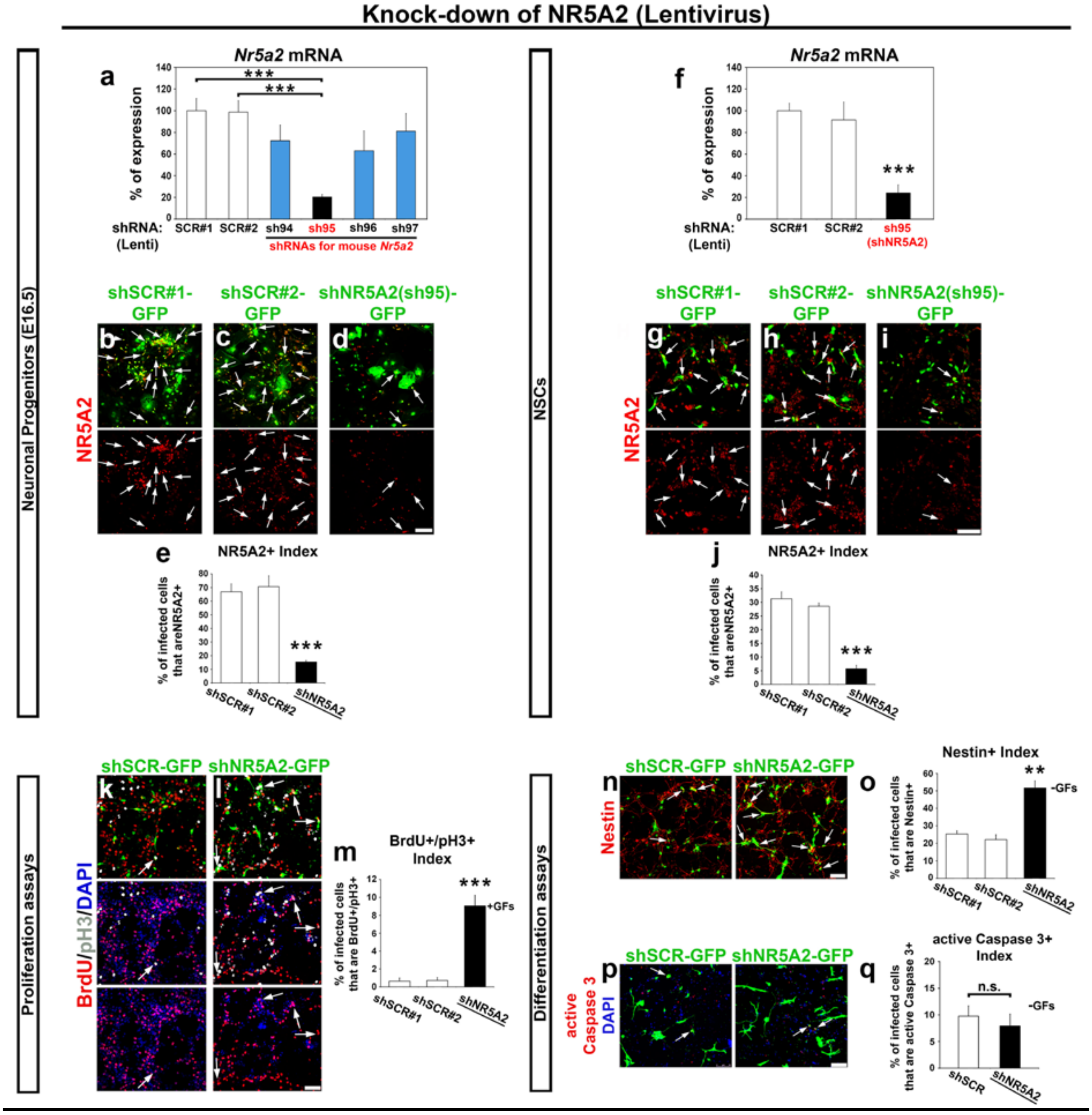

<u>Supplementary Figure 4</u>. Knock-down analysis of NR5A2 in spinal cord-derived NSCs.

(**a**) RT-qPCR analysis indicating the relative expression levels of *Nr5a2* mRNA in neuronal progenitors isolated from *E16.5* mouse spinal cord and infected with different lentiviruses encoding shRNAs targeting mouse *Nr5a2* (sh94-97) or control-scrambled sequences (shSCR#1, shSCR#2).

(**b-e**) Double immunolabelings of GFP (green) with NR5A2 (red) in neuronal progenitors infected with shSCRs-GFP (b-c) or shNR5A2-GFP (sh95) (d), as indicated. Quantification of NR5A2 index is shown in (e) (% of GFP+; NR5A2+ / total GFP+).

(**f-g**) RT-qPCR (f) and double GFP (green)/NR5A2 (red) immunofluorescence analysis (g-i) confirming the down-regulation of *Nr5a2* mRNA and protein levels, respectively, in NSCs that were infected with a lentivirus encoding the distinct and potent shRNA against NR5A2 (sh95) compared to control lentiviruses (shSCR#1, shSCR#2). Quantification of NR5A2 index is shown in (j).

(**k-l**) Triple immunofluorescence analysis of GFP (green), BrdU (red) and pH3 (grey, artificially pseudo-colored after confocal microscopy analysis) in proliferating NSCs infected with shSCR-GFP (k) or shNR5A2-GFP (l) and pulsed for 2 h with BrdU.

(**m**) Quantification of the GFP+ NSCs that are double positive for BrdU and pH3.

(**n-o**) Double GFP (green)/Nestin (red) immunostainings of infected NSCs cultured in the absence of GFs (n) and quantification of the knock-down experimental data (o).

(**p-q**) Double GFP (green)/active Caspase 3 (red) immunostainings of NSCs infected with shSCR-GFP or shNR5A2-GFP lentiviruses (p) and quantification of apoptosis (q).

In each case, arrows indicate GFP+ cells that are co-localized with each marker. The results are shown as mean ±SD. n.s.: not significant $P>0.05$, ** $P<0.01$, *** $P<0.001$ (Student's *t*-test). Scale bars: (b-d, g-i) 100 μm; (k-l, n, p) 50 μm.

Supplementary Figure 5

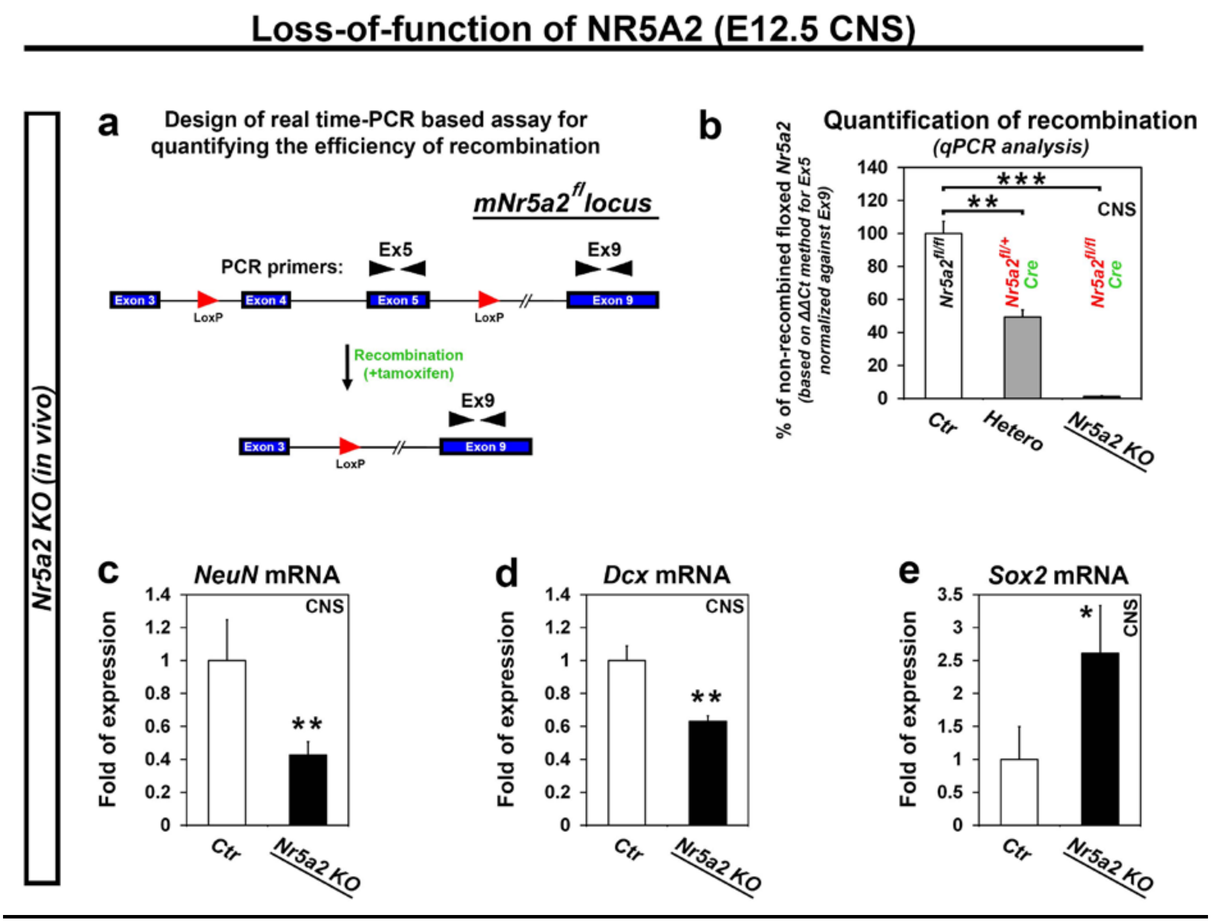

Supplementary Figure 5. Loss-of-function analysis of NR5A2 in *E12.5* CNS.

(**a**) Design of real time-PCR based assay for quantifying the efficiency of recombination of the floxed *Nr5a2* locus (based on ΔΔCt method for *exon5* normalized to *exon9*).

(**b**) Quantification of recombination efficiency of the floxed *Nr5a2* locus by qPCR analysis, as indicated (% of non-recombined floxed *Nr5a2*).

(**c-e**) RT-qPCRs showing the quantifications of *NeuN* (c), *DCX* (d), and *Sox2* (e) mRNA levels in *E12.5* CNS of *Ctr* and *Nr5a2 KO* animals.

All values represent the mean ±SD of four animals (n=4). * P<0.05, ** P<0.01, *** P<0.001 (Student's t-test).

Supplementary Figure 6

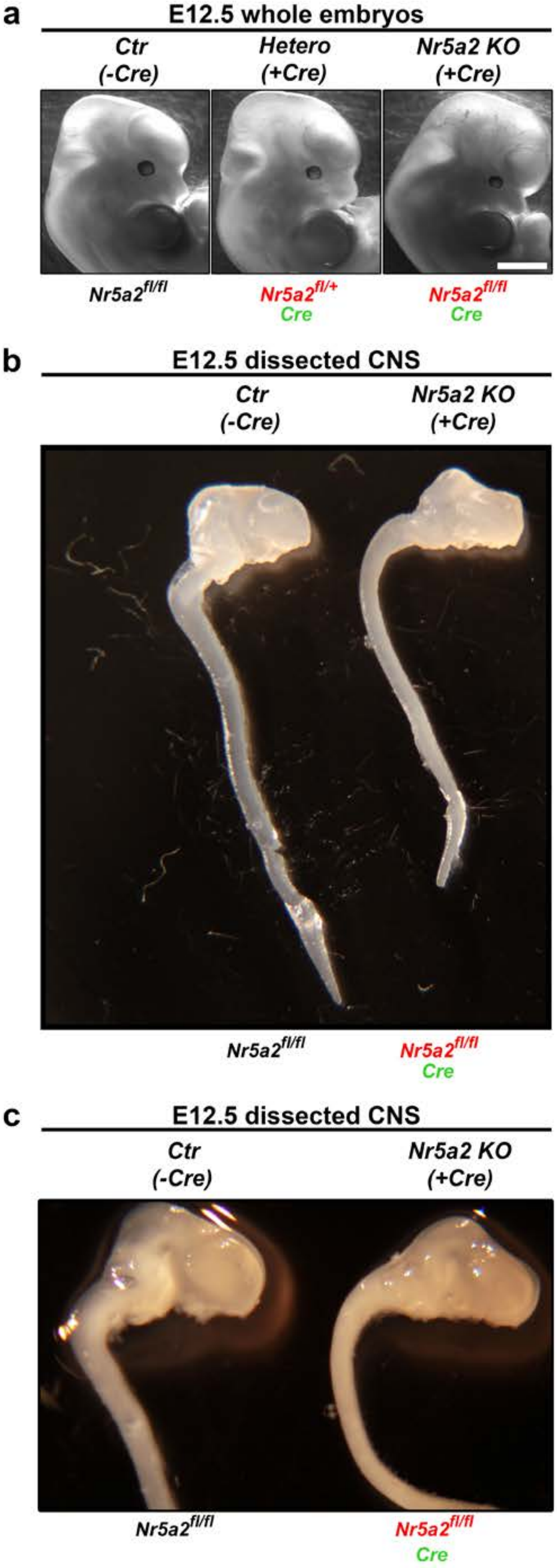

Supplementary Figure 6. Inducible deletion of *Nr5a2* causes severe size reduction of spinal cord and brain in *E12.5* mouse embryos.

(**a**) Larger magnifications of *Ctr* (*Nr5a2*$^{fl/fl}$*;without CreER*) (left panel), *Hetero* (*Nr5a2*$^{fl/+}$*;CreER*) (center panel) and *Nr5a2 KO* (*Nr5a2*$^{fl/fl}$*;CreER*) (right panel) *E12.5* whole embryos stereoscopically depicted in Fig. 4e. Scale bar: 250 μm.

(**b and c**) Colored photographs of dissected *E12.5* experimental mouse CNS. Note the large difference in the size of the CNS (spinal cord and brain) of *Nr5a2 KO* mice.

(b) and (c) photographs were separately captured and depict the same *Ctr* and *Nr5a2 KO* dissected CNS.

Supplementary Figure 7

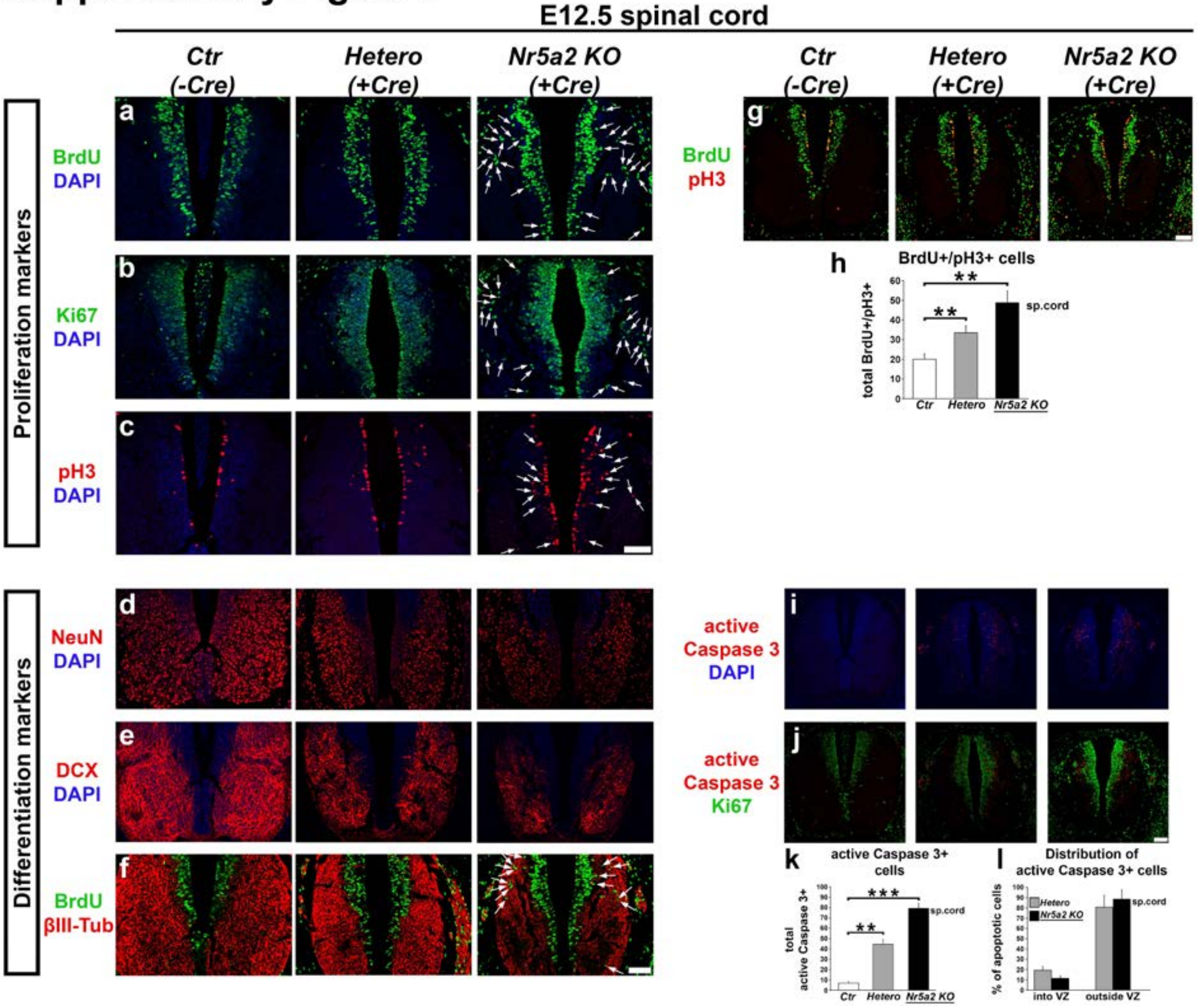

<u>Supplementary Figure 7</u>. Cre/LoxP-mediated deletion of NR5A2 results in increased numbers of proliferating NSCs and enhanced cell cycle progression during spinal cord development.

(**a-f**) Larger magnifications of *Ctr* (*Nr5a2*$^{fl/fl}$*;without CreER*) (left panels), *Hetero* (*Nr5a2*$^{fl/+}$*;CreER*) (center panels) and *Nr5a2 KO* (*Nr5a2*$^{fl/fl}$*;CreER*) (right panels) *E12.5* spinal cords depicted in Figure 4h-4q. Arrows mark the ectopic proliferating cells [BrdU+ (a), Ki67+ (b), pH3+ (c)] that are localized outside the VZ and do not express neuronal markers [e.g. βIII-Tubulin- (f)].

(**g**) Double immunostainings of BrdU (green) with pH3 (red) in mouse embryos pulsed for 2 h with BrdU.

(**h**) Quantification of double BrdU+/pH3+ cells (total double+ / spinal cord section).

(**i-j**) Double immunolabelings of active Caspase 3 (red) with Ki67 (green) on sections from *E12.5* spinal cord.

(**k**) Quantification of apoptotic cells (active Caspase 3+) in *Ctr*, *Hetero* and *Nr5a2 KO* embryos (total active Caspase 3+ / spinal cord section).

(**l**) Distribution of active Caspase+ cells between VZ and outside VZ (% of apoptotic cells), according to the proliferation marker Ki67.

Note that immunofluorescence images in (a), (f) and (g) depict the same spinal cord sections from *Ctr*, *Hetero* and *Nr5a2 KO* embryos, labeled with triple-antibody staining (BrdU/pH3/βIII-Tubulin) and pseudo-colored as green (BrdU) and red (pH3 or βIII-Tubulin) after confocal microscopy analysis.

All values represent the mean ±SD of four animals (n=4). Cell nuclei were visualized with DAPI staining (blue). ** P<0.01, *** P<0.001 (Student's *t*-test). Scale bars: 75 µm.

Supplementary Figure 8

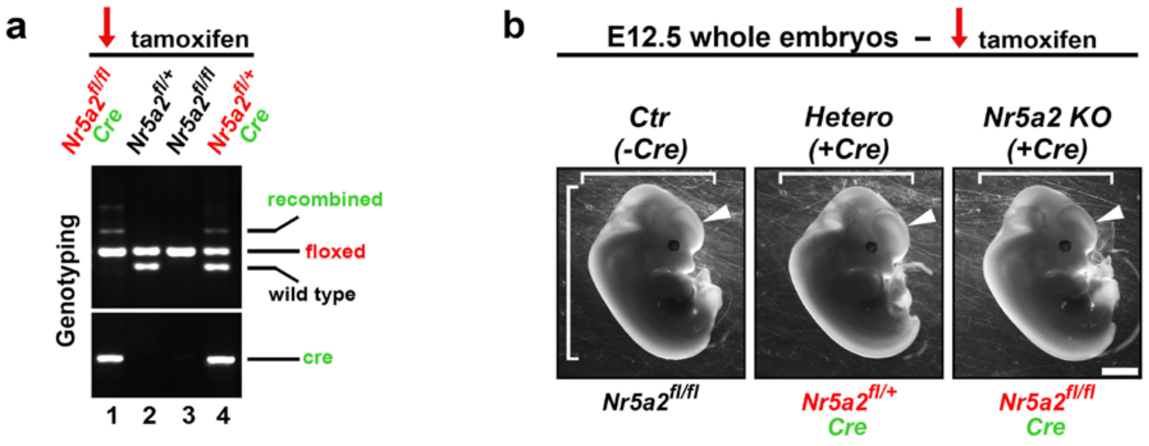

<u>Supplementary Figure 8</u>. Control experiment with low dose of tamoxifen of the Cre/LoxP-mediated recombination.

(**a**) Assessment of recombination of the floxed *Nr5a2* locus by genotyping PCR in CNS tissue, under conditions of minimal dose of tamoxifen, as indicated. Note the non-efficient recombination (green) of the floxed *Nr5a2* alleles despite the presence of a single allele of *CreER* (*lane 1*: homozygous floxed alleles; *lane 4*: heterozygous alleles) (compare Supplementary Fig. 8a with Fig. 4b).

(**b**) Stereoscopic views of *E12.5 Ctr*, *Hetero* and *Nr5a2 KO* whole mouse embryos that received minimal dose of tamoxifen. The white arrowheads depict the forebrain of each representative embryo. No defects were observed.

Scale bar: 250 µm.

Supplementary Figure 9

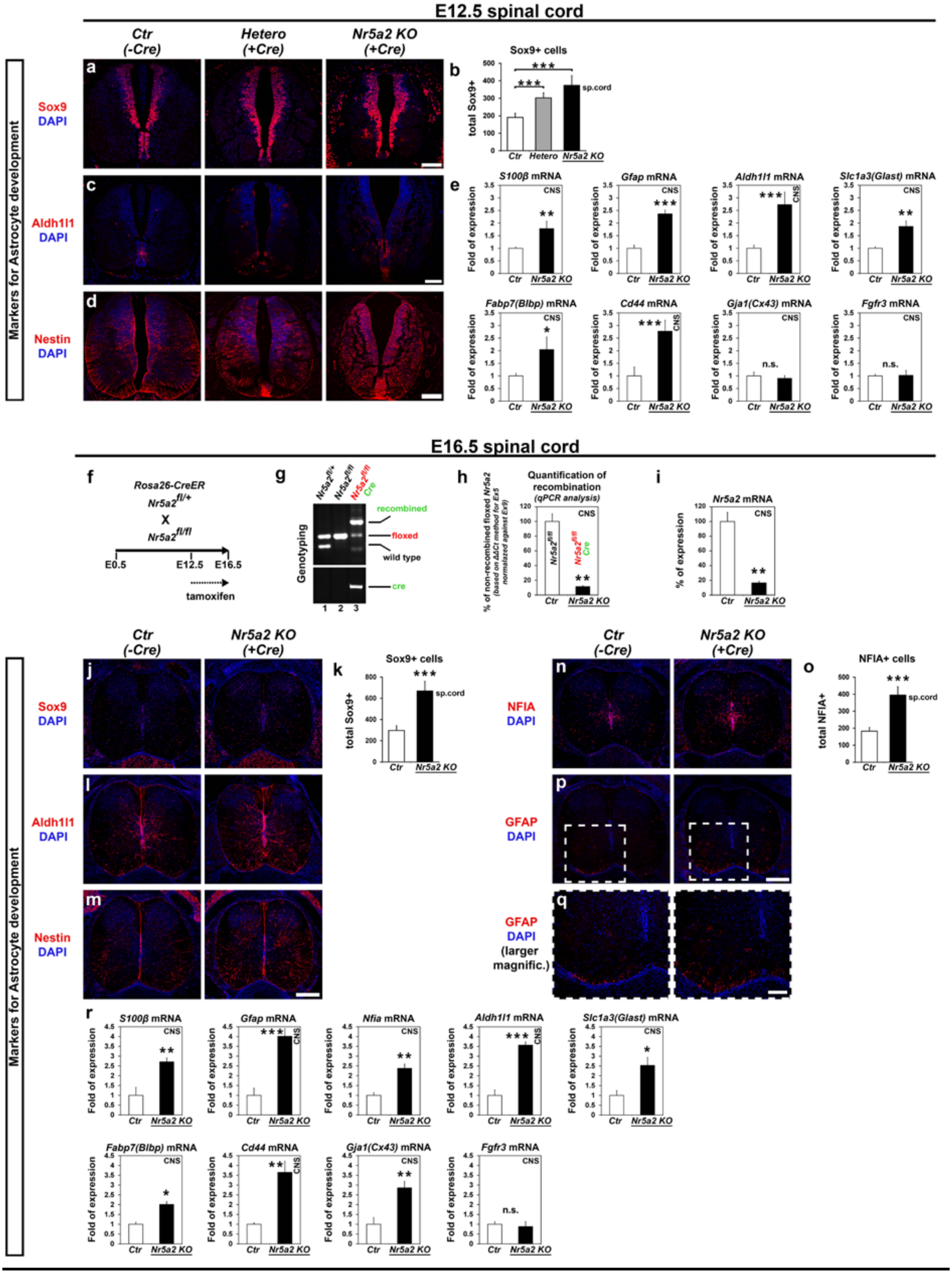

Supplementary Figure 9. Temporal deletion of *Nr5a2* affects astrocyte development.

(**a-d**) Phenotypic analysis of *Hetero* (center panels) and *Nr5a2 KO* (right panels) *E12.5* spinal cords, compared to *Ctr* (left panels), in relation to various markers for astrocyte development: (a-d) Immunofluorescence images of representative sections depicting Sox9+ (a), Aldh1l1+ (c) and Nestin+ (d) cells (red), as indicated. Note the ectopic Sox9+ cells in (a). Quantification of Sox9+ cells is shown in (b) (total Sox9+ / spinal cord section).

(**e**) RT-qPCR analyses in *E12.5* CNS of *Ctr* and *Nr5a2 KO* animals.

(**f**) Schematic representation of the *Nr5a2* knock-out strategy during later stages of development (*E12.5* to *E16.5*).

(**g**) Assessment of recombination of the floxed *Nr5a2* locus by genotyping PCR in *E16.5* CNS tissue, as indicated. Note the efficient recombination of the floxed *Nr5a2* alleles in the presence of a single allele of *CreER* (*lane 3*).

(**h**) Quantification of recombination efficiency of the floxed *Nr5a2* locus by qPCR analysis in *E16.5* CNS tissue, as indicated (% of non-recombined floxed *Nr5a2*).

(**i**) RT-qPCR for the detection of *Nr5a2* mRNA levels in *E16.5* CNS of *Ctr* and *Nr5a2 KO* animals.

(**j-q**) Phenotypic analysis of *Nr5a2 KO* (right panels) *E16.5* spinal cords, compared to *Ctr* (left panels), in relation to various markers for astrocyte development: (j-q) Immunofluorescence images of representative sections depicting Sox9+ (j), Aldh1l1+ (l), Nestin+ (m), NFIA+ (n) and GFAP+ (p-q) cells (red), as indicated. (q) micrographs are larger magnifications of the square shapes depicted in (p). Quantifications of Sox9+ and NFIA+ cells are shown in (k) and (o), respectively (total Sox9+ or NFIA+ / spinal cord section).

(**r**) RT-qPCR analyses in *E16.5* CNS of *Ctr* and *Nr5a2 KO* animals.

All values represent the mean ±SD of four animals (n=4). Cell nuclei were visualized with DAPI staining (blue). n.s.: not significant $P>0.05$, * $P<0.05$, ** $P<0.01$, *** $P<0.001$ (Student's *t*-test). Scale bars: (a-d) 100 μm; (j-p) 250 μm; (q) 100 μm.

Supplementary Figure 10

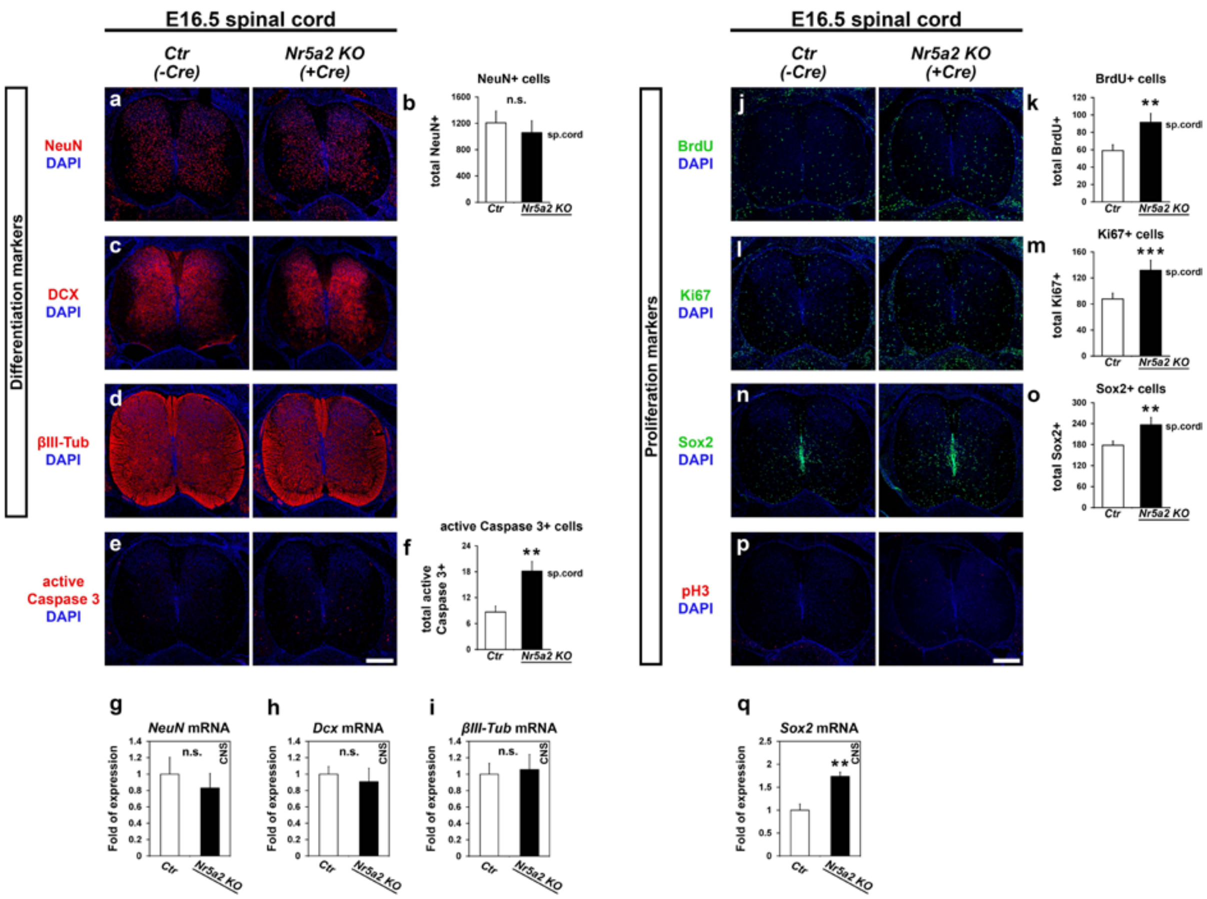

<u>Supplementary Figure 10</u>. Cre/LoxP-mediated deletion of NR5A2 provokes a slight increase in numbers of proliferating cells without affecting neuronal differentiated-cells during later stages of spinal cord development (*E16.5*).

(**a-f**) Phenotypic analysis of *Nr5a2 KO* (right panels) *E16.5* spinal cords, compared to *Ctr* (left panels), in relation to various differentiation markers: (a-d) Immunofluorescence images of transverse paraffin representative sections depicting NeuN+ (a), DCX+ (c) and βIII-Tubulin+ (d) cells (red). Active caspase 3+ staining is depicted in (e) (red). Quantifications of NeuN+ and active caspase 3+ cells are shown in (b) and (f), respectively (total NeuN+ or active caspase 3+ / spinal cord section).

(**g-i**) RT-qPCRs showing the quantifications of *NeuN* (g), *DCX* (h) and *βIII-Tubulin* (i) mRNA levels in *E16.5* CNS of *Ctr* and *Nr5a2 KO* animals, as indicated.

(**j-p**) Phenotypic analysis of the same embryos, in relation to various proliferation markers: (j-p) Immunofluorescence images of representative spinal cord sections depicting BrdU+ (j), Ki67+ (l), Sox2+ (n) (green) and pH3+ (p) (red) cells. (j) Mouse embryos (*E16.5*) were pulsed for 3 h with BrdU and then labeled with the appropriate anti-BrdU primary antibody. Quantifications of BrdU+, Ki67+ and Sox2+ cells are shown in (k), (m) and (o), respectively (total marker+ / spinal cord section).

(**q**) RT-qPCR showing the quantification of *Sox2* mRNA levels.

All values represent the mean ±SD of four animals (n=4). Cell nuclei were visualized with DAPI staining (blue). n.s.: not significant $P>0.05$, ** $P<0.01$, *** $P<0.001$ (Student's *t*-test). Scale bars: 250 μm.

Supplementary Figure 11

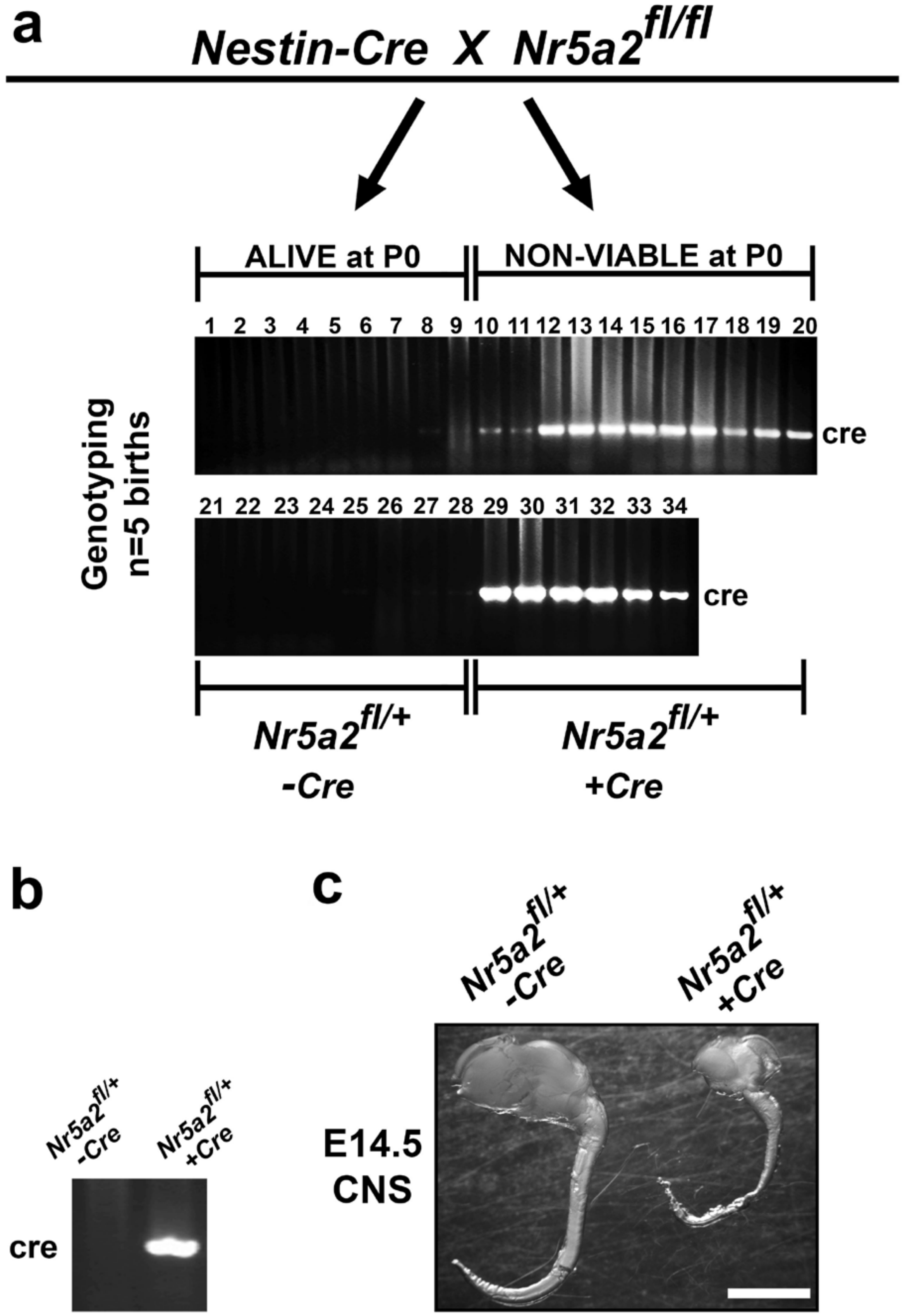

<u>Supplementary Figure 11</u>. Conditional inactivation of NR5A2 from *Nestin*+ NSCs (*Nestin-Cre* X *Nr5a2*$^{fl/fl}$ mice) causes severe CNS size reduction and lethality around birth.

(**a**) Genotyping by PCR from mouse tail biopsies of newly-born embryos (34 *P0* mice, n=5 births). Note that all non-viable mice (10-20, 29-34; right parts of the gels) were carrying the *Nestin-Cre* allele (*Nr5a2*$^{fl/+}$;*Nestin-Cre*) while all alive mice (1-9, 21-28; left parts of the gels) were negative for this allele (*Nr5a2*$^{fl/+}$*;without Nestin-Cre*).

(**b and c**) Genotyping PCR (b) and stereoscopic views of *E14.5* experimental mouse CNS (c). Note the large difference in the size of the CNS (brain and spinal cord) of *Nr5a2*$^{fl/+}$;*Nestin-Cre* mice.

All PCR reaction mixtures included *Cre*-specific primers to amplify the corresponding DNA sequences and were analyzed on 2% agarose gels. Scale bar: 250 μm.

Supplementary Figure 12

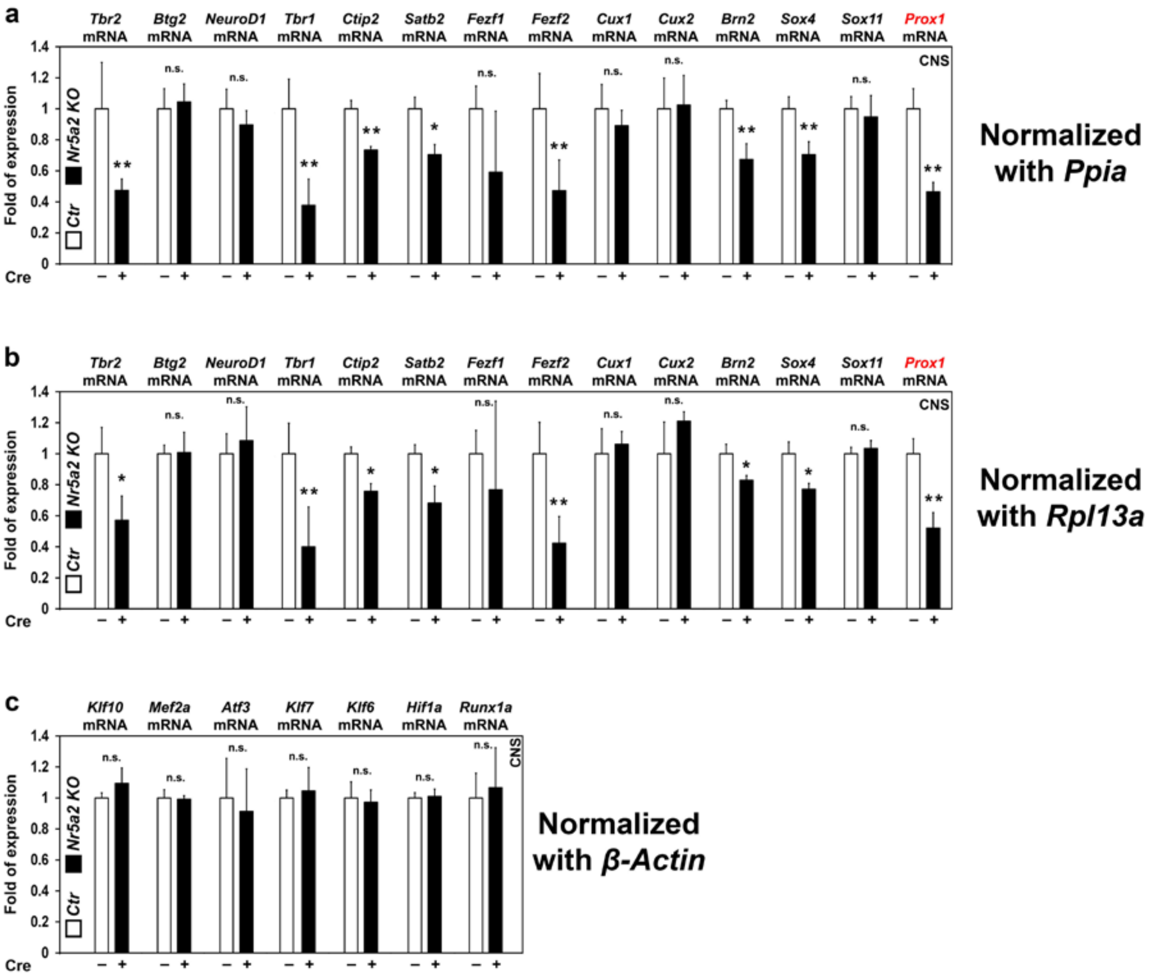

<u>Supplementary Figure 12</u>. RT-qPCR analyses in *E12.5* CNS of *Ctr* and *Nr5a2 KO* embryos.

(**a and b**) RT-qPCR analyses indicating the relative expression mRNA levels of a number of genes critical for neuronal differentiation, as indicated. Measured values were normalized using *Ppia* (a) or *Rpl13a* (b) mRNA levels as internal references (compare Supplementary Fig. 12a-12b with Fig. 6a).

(**c**) RT-qPCR quantifications of a subset of genes encoding for transcription factors which are not directly related to neuronal differentiation, namely *Klf10*, *Mef2a*, *Atf3*, *Klf7*, *Klf6*, *Hif1a* and *Runx1a*, as indicated. Measured values were normalized using *β-actin* mRNA as internal reference.

All values represent the mean ±SD of four animals (n=4). n.s.: not significant $P>0.05$, * $P<0.05$, ** $P<0.01$ (Student's *t*-test).

Supplementary Figure 13

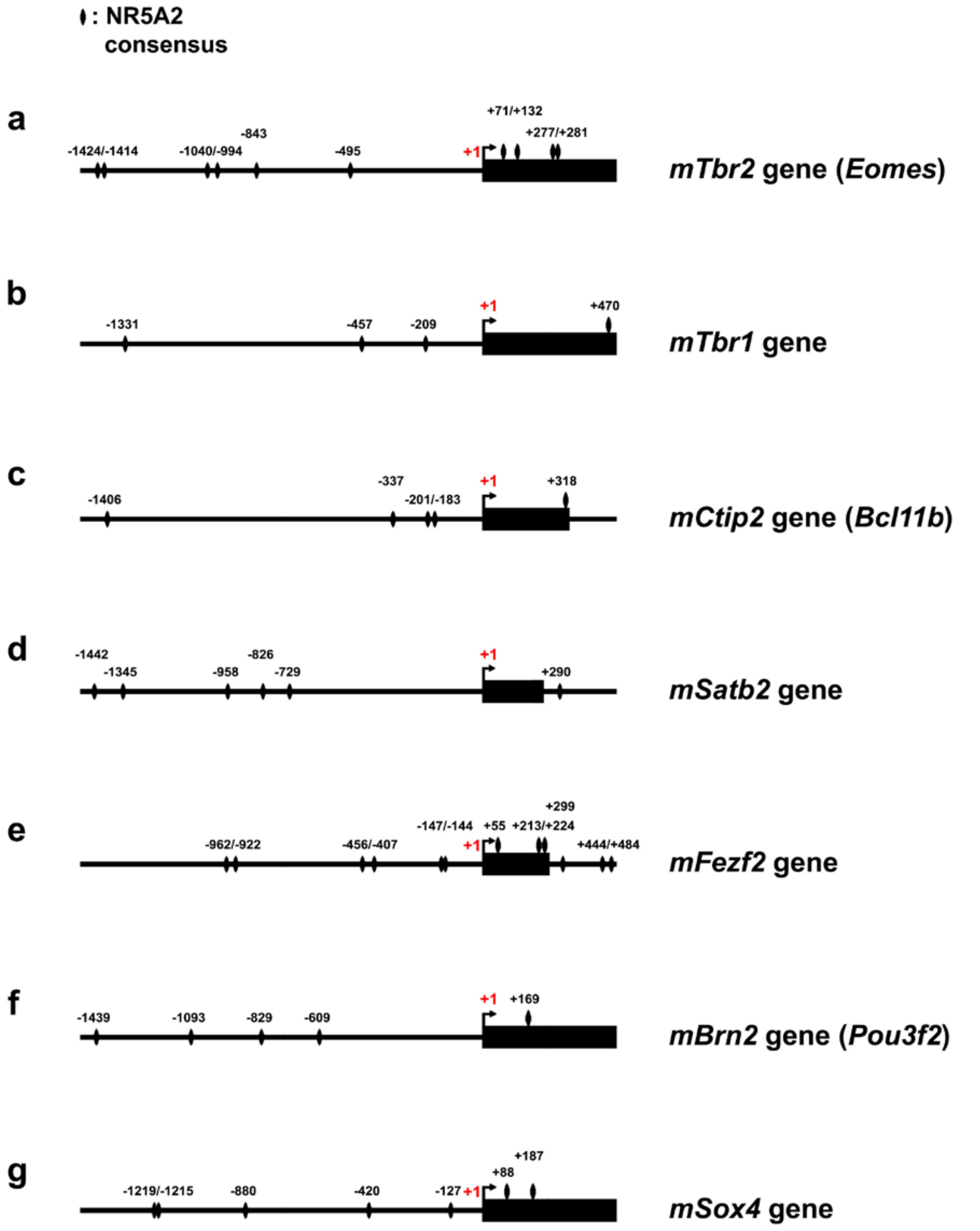

Supplementary Figure 13. Identification of NR5A2 consensus elements in the genomic loci of NR5A2-affected genes.

(**a-g**) Bioinformatic promoter analysis of a number of NR5A2-affected genes critical for neuronal differentiation [*Tbr2* (a), *Tbr1* (b), *Ctip2* (c), *Satb2* (d), *Fezf2* (e), *Brn2* (f) and *Sox4* (g)], as indicated.

Promoter sequences from mouse genome were downloaded from publicly available data (DBTSS-DataBase of Transcriptional Start Sites and confirmed in UCSC genome browser) and correspond from -500 bp downstream to +1,500 bp upstream of the genes' TSSs based on transcription start annotations. The consensus binding sequences of NR5A2 in each gene are depicted.

Supplementary Figure 14

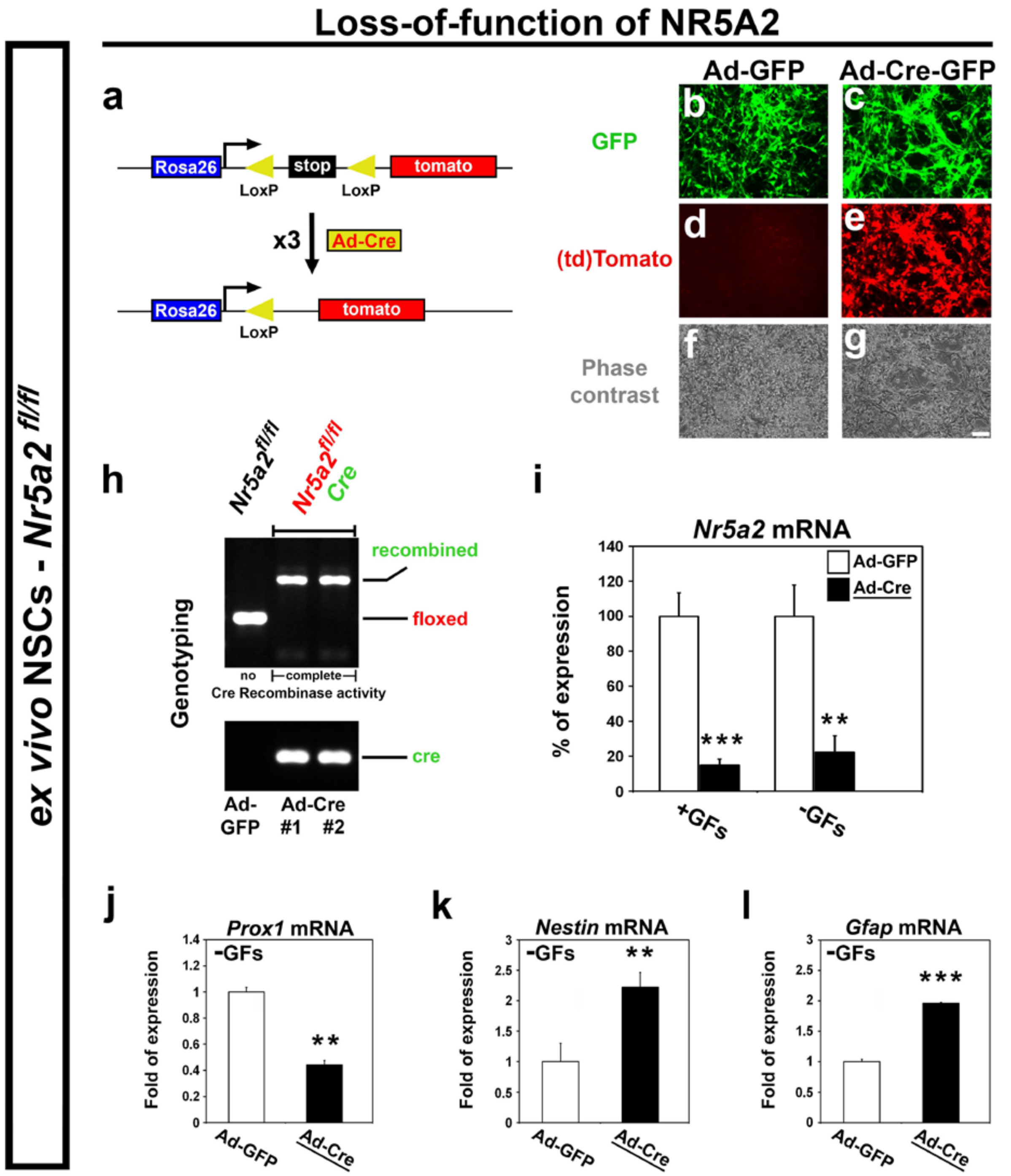

__Supplementary Figure 14__. **Adenovirus-mediated genetic deletion of NR5A2 from NSCs is sufficient to substantially diminish *Prox1* expression.**

(**a**) Schematic representation of the (td)Tomato reporter cassette (red) knocked-into the *Rosa26 locus* (blue) by homologous recombination. A LoxP-floxed STOP cassette is located

upstream of the *(td)Tomato* gene, thus preventing expression of the red-emitting fluorophore when Cre recombinase is absent. In the presence of Cre recombinase (+Ad-Cre), the STOP sequence between the LoxP sites (yellow) is excised, thus leading to permanent and constitutive expression of the (td)Tomato protein in NSCs.

(**b-g**) Fluorescent microscope images of GFP (green) (b-c) or (td)Tomato (red) (d-e) in *Rosa26-(td)Tomato* NSCs isolated from *E14.5* spinal cords and transduced three times (x3) with GFP alone- (Ad-GFP) or Cre-expressing adenoviruses (Ad-Cre-GFP). Phase contrast images of cells are shown in (f-g). Nearly all NSCs were efficiently transduced, as indicated by the massive expression of (td)Tomato following Cre-mediated recombination.

(**h**) Assessment of recombination of floxed *Nr5a2* locus by PCR amplification of genomic DNAs extracted from *Nr5a2*$^{fl/fl}$ NSCs that were transduced three times with Ad-GFP or Ad-Cre, as indicated. Note the complete recombination (green) of the floxed *Nr5a2* alleles in the presence of a single allele of *Cre* (Ad-Cre#1, #2) (compare Supplementary Fig. 14h with Fig. 4b).

(**i**) RT-qPCRs showing the quantifications of *Nr5a2* relative mRNA levels in proliferating (+GFs) or differentiating (-GFs) *Nr5a2*$^{fl/fl}$ NSCs that were infected with adenoviruses (Ad-GFP, Ad-Cre).

(**j-l**) RT-qPCR analyses for the detection of *Prox1* (j), *Nestin* (k) and *Gfap* (l) mRNA levels in transduced *Nr5a2*$^{fl/fl}$ NSCs upon withdrawal of GFs, as indicated.

The results are shown as mean ±SD. ** P<0.01, *** P<0.001 (Student's t-test). Scale bar: 50 µm.

Supplementary Figure 15

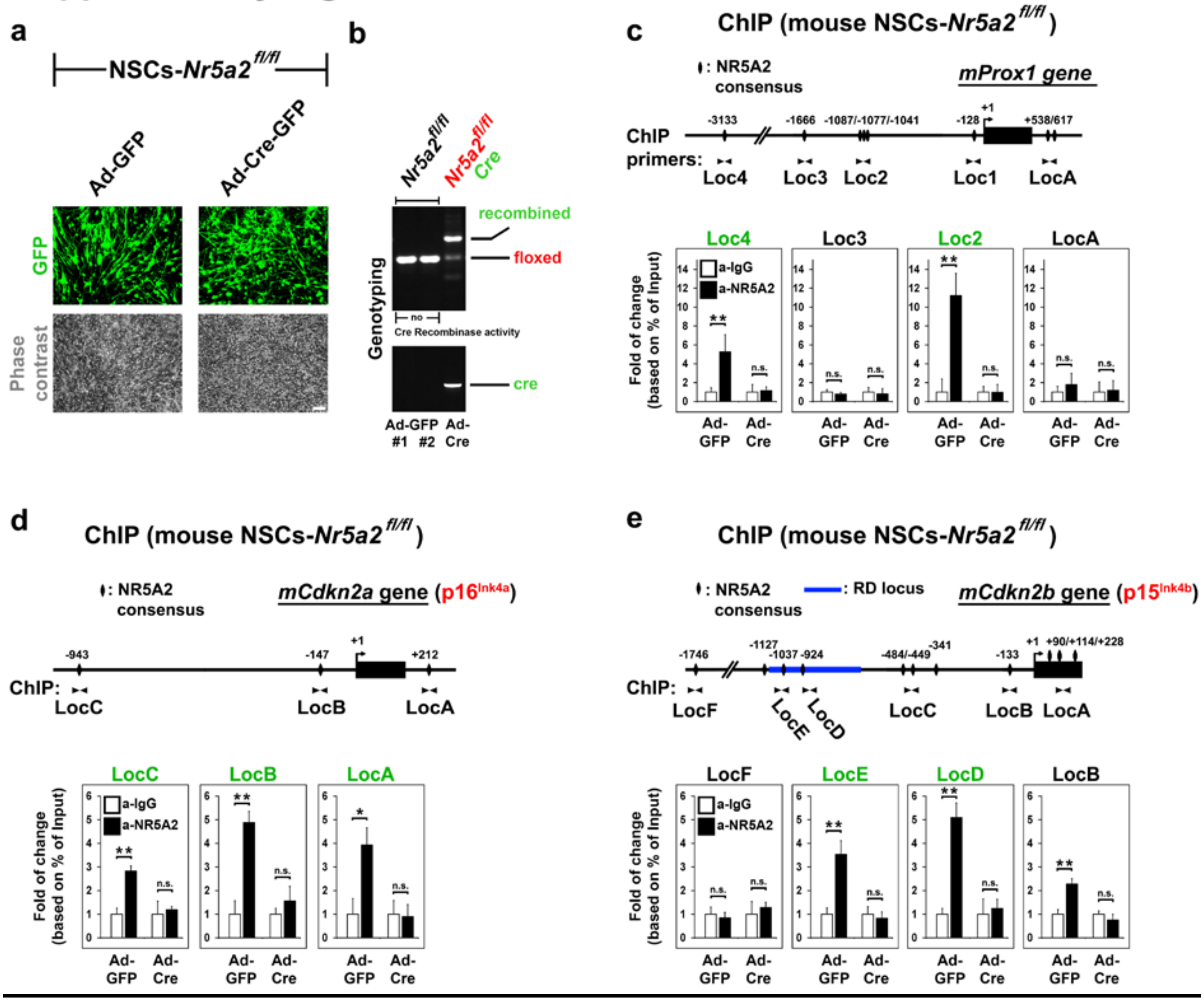

<u>Supplementary Figure 15</u>. Genetic deletion of *Nr5a2* from NSCs is sufficient to abolish the recruitment of NR5A2 to the promoters of *Prox1*, *p16^{Ink4a}* and *p15^{Ink4b}* genes.

(**a**) Fluorescent microscope images of GFP (green) in *Nr5a2$^{fl/fl}$* NSCs isolated from *E14.5* spinal cords and transduced three times (x3) with GFP alone- (Ad-GFP) or Cre-expressing adenoviruses (Ad-Cre-GFP). Phase contrast images of *ex vivo* cultured cells are also shown. Nearly all NSCs were efficiently transduced.

(**b**) Assessment of recombination of floxed *Nr5a2* locus by PCR amplification of genomic DNAs extracted from *Nr5a2$^{fl/fl}$* NSCs that were transduced three times with Ad-GFP or Ad-Cre, as indicated. Note the efficient recombination (green) of the floxed *Nr5a2* alleles in the presence of a single allele of *Cre* (Ad-Cre) (compare with Supplementary Fig. 14h).

(**c-e**) Schematics of the organization of mouse *Prox1* (c), *Cdkn2a* (d) and *Cdkn2b* (e) gene loci (upper panels). The lower panels present ChIP analyses for NR5A2 binding in chromatin samples prepared from *Nr5a2*$^{fl/fl}$ NSCs that were transduced with Ad-GFP or Ad-Cre. The specific consensus sites that NR5A2 is directly recruited to, are represented in green. Following Cre/LoxP-mediated deletion of NR5A2 (+Ad-Cre), the enrichments at the specific loci are abolished.

The results are shown as mean ±SD. n.s.: not significant P>0.05, * P<0.05, ** P<0.01 (Student's *t*-test). Scale bar: 50 μm.

Supplementary Figure 16

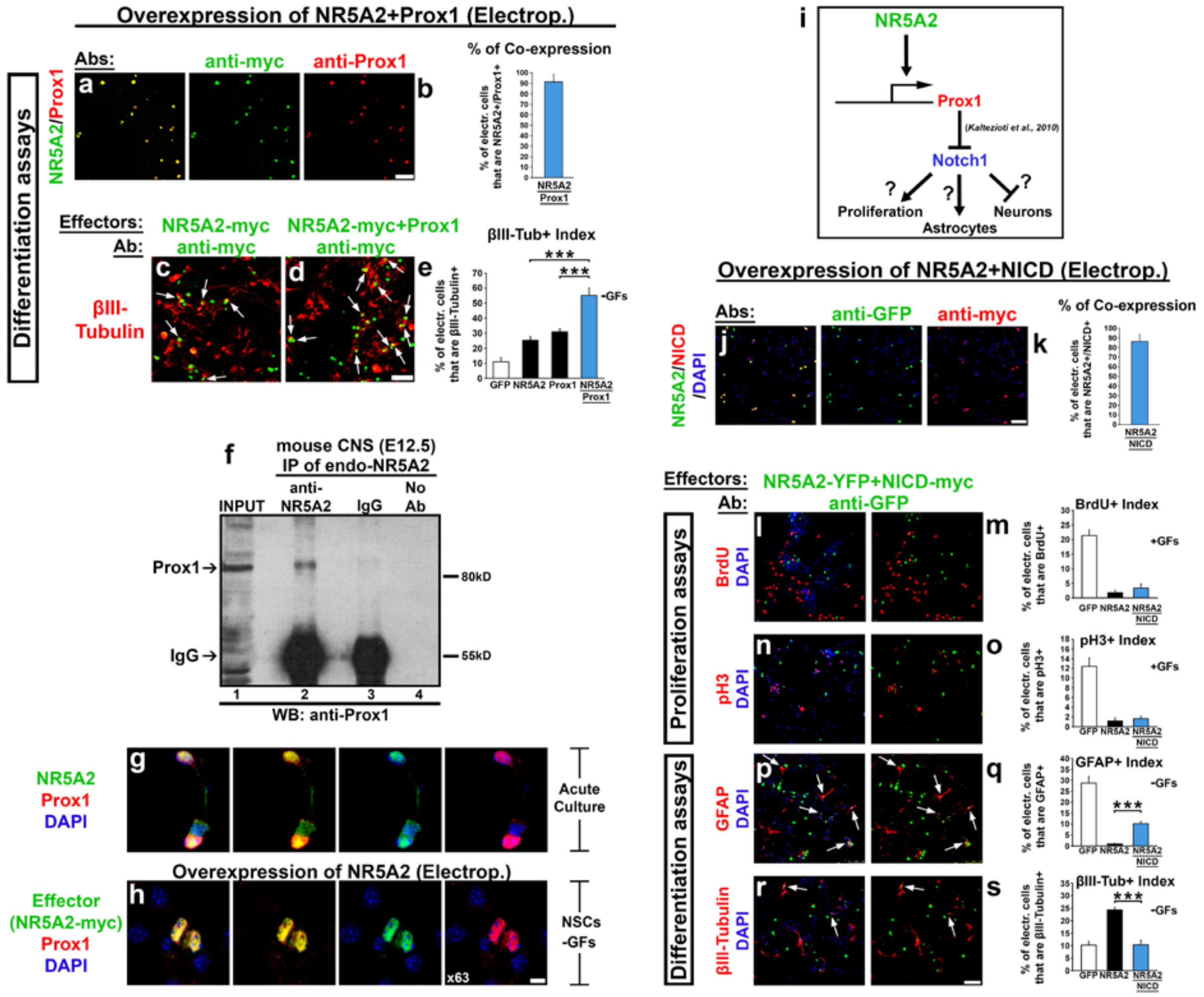

<u>Supplementary Figure 16</u>. NR5A2 functionally and directly interacts with Prox1 protein to synergistically control the Notch1-mediated suppression of neurogenesis.

(**a-e**) Double immunostainings of myc (green) with Prox1 (red) (a) or βIII-Tubulin (red) (c-d) in NSCs co-electroporated with NR5A2-myc+Prox1 expression vectors (a, d) and cultured under differentiating conditions. Arrows indicate representative double positive cells. The percentage of electroporated cells that are double NR5A2+/Prox1+ is presented in (b). Quantification of βIII-Tubulin index is shown in (e) (% of GFP+ or NR5A2-myc+ or Prox1+ or NR5A2-myc+Prox1+; βIII-Tubulin+ / total GFP+ or NR5A2-myc+ or Prox1+ or NR5A2-myc+Prox1+).

(**f**) NR5A2 binds Prox1 *in vivo*: Cell lysates from WT mouse embryonic CNS (*E12.5*) were subjected to immunoprecipitations with anti-NR5A2 antibody (*lane 2*) or control anti-rabbit IgGs (*lane 3*), followed by western immunoblotting with anti-Prox1 antibody. The positions of Prox1 and heavy chain of IgGs are shown on the left.

(**g-h**) Double immunolabelings of NR5A2 or myc (green) with Prox1 (red) in untreated *acute* cells (g) or NR5A2-myc electroporated NSCs cultured in the absence of GFs (h), respectively. Images were obtained with a x63 oil immersion objective on a Leica confocal microscope.

(**i**) Schematic representation of the possible role of NR5A2 in inducing Prox1-mediated suppression of Notch1 pathway that eventually leads to enhanced neurogenesis[1].

(**j-s**) Double immunostainings of GFP (green, detects YFP) with myc (red) (j) or various markers [BrdU (l), pH3 (n), GFAP (p) and βIII-Tubulin (r)] (all red) in NSCs co-electroporated with NR5A2-YFP+NICD-myc expression vectors and cultured in the presence (l-n) or absence of GFs (p-r), as indicated. Arrows indicate the double NR5A2-YFP+/NICD-myc+ cells that co-express GFAP (p) or βIII-Tubulin (r) differentiation markers. Quantifications of co-expression and the indices of the above markers are shown in k, m, o, q and s, respectively (% of GFP+ or NR5A2-YFP+ or NR5A2-YFP+NICD-myc+; marker+ / total GFP+ or NR5A2-YFP+ or NR5A2-YFP+NICD-myc+).

Cell nuclei were visualized with DAPI staining (blue). The results are shown as mean ±SD. *** $P<0.001$ (Student's *t*-test). Scale bars: (a, c-d) 50 μm; (g-h) 7.5 μm; (j-r) 75 μm.

Supplementary Figure 17

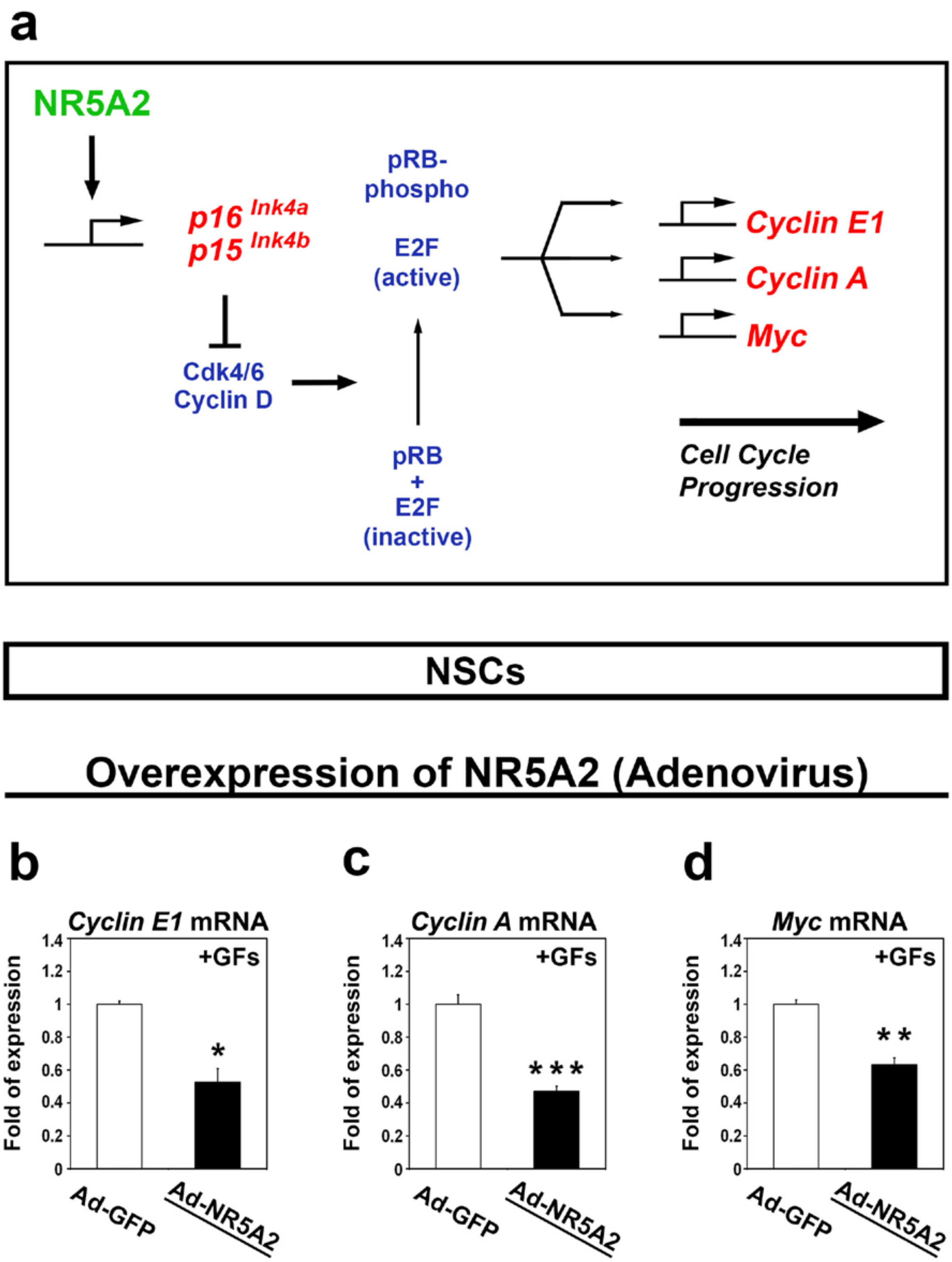

Supplementary Figure 17. NR5A2 overexpression suppresses downstream targets of p16^{Ink4a} and p15^{Ink4b} pathway.

(**a**) Schematic representation of the role of NR5A2 in arresting cell cycle progression of NSCs through induction of *p16*^{Ink4a} and *p15*^{Ink4b} and subsequent down-regulation of E2F/phospho-pRb-dependent cell cycle-promoting genes (*Cyclin E1*, *Cyclin A* and *Myc*).

(**b-d**) RT-qPCR analysis indicating the relative expression levels of *Cyclin E1* (b), *Cyclin A* (c) and *Myc* (d) mRNAs in NSCs infected with GFP- (Ad-GFP) or NR5A2- (Ad-NR5A2) expressing adenoviruses and cultured in the presence of GFs.

The results are shown as mean ±SD. * P<0.05, ** P<0.01, *** P<0.001 (Student's *t*-test).

Supplementary Figure 18

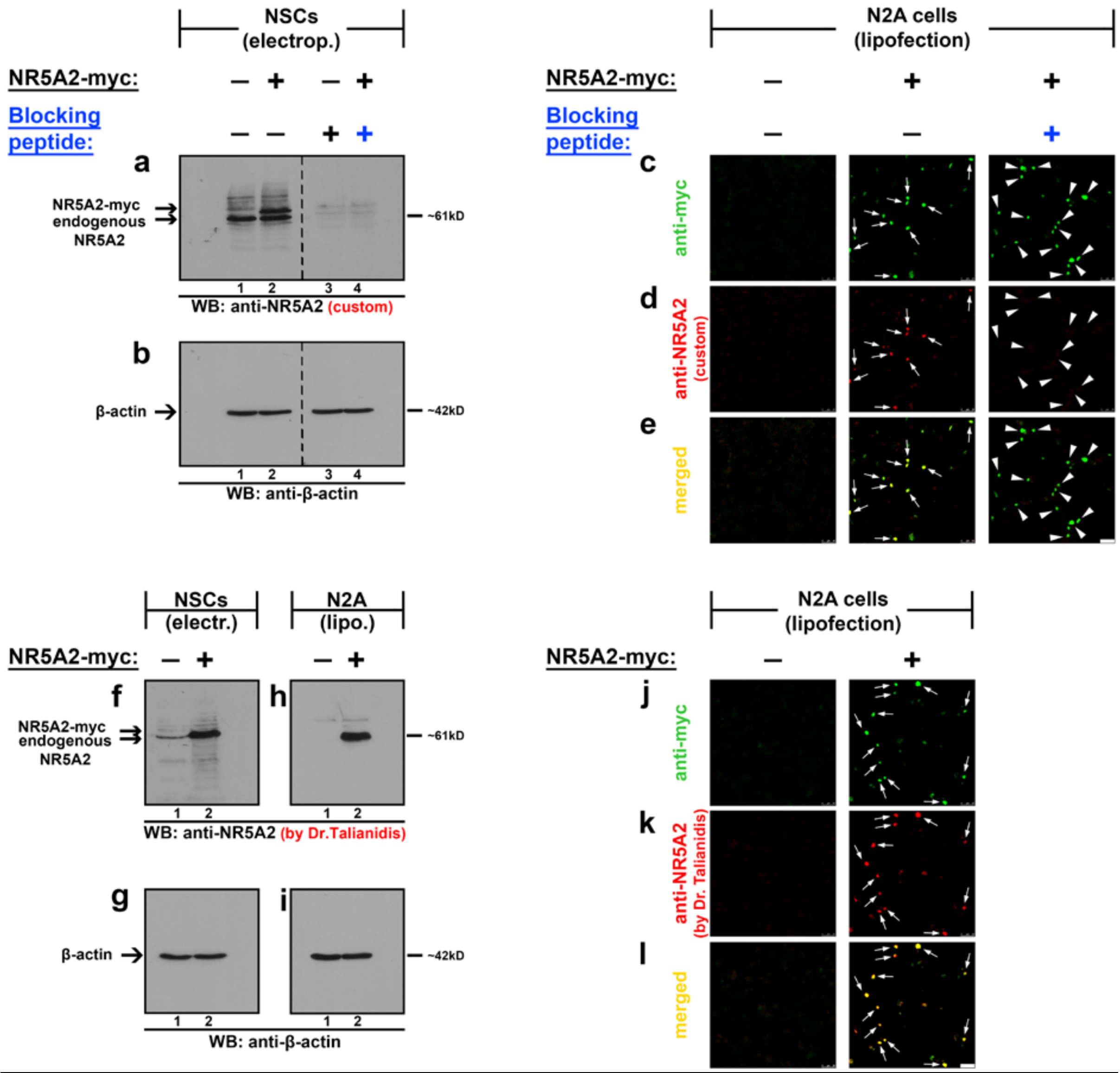

<u>Supplementary Figure 18</u>. Quality and specificity test of the rabbit polyclonal anti-NR5A2 antibodies that were used in this study.

(**a-e**) The anti-NR5A2 custom-made antibody was raised against mouse NR5A2 peptide (reported in *Methods*) and produced by Davids Biotechnologie GmbH: Western blot (a-b) and immunofluorescence (c-e) analyses in NSCs and N2A cells, respectively. NSCs or N2A were electroporated or lipofected, respectively, with NR5A2-myc (+) or pcDNA3 (-) expression vectors, as indicated. Pre-absorption of the antiserum with the corresponding immunogen peptide (blue color-*blocking* peptide) completely abolished the western blot bands (a; right

part of gel) and the immunostaining [arrowheads in (d)] (*peptide competition assays*). Please note that the membrane shown in (a) and (b) was cut in the middle and its right part was probed with the anti-NR5A2 antibody together with the *blocking* peptide, as indicated.

(**f-l**) NR5A2 protein was detected using a rabbit polyclonal anti-NR5A2 antibody (kindly donated by Dr. I. Talianidis): Western blot (f-i´) and immunofluorescence (j-l) analyses in NSCs and N2A cells, as indicated. NSCs or N2A cells were electroporated or lipofected, respectively with NR5A2-myc (+) or pcDNA3 (-) expression vectors, as indicated. Immunogen peptide was not available for this antibody.

Exogenous and endogenous western blot bands of NR5A2 shown in (a), (f), and (h) are marked on the left. Protein loads were verified with β-actin as reference protein, using a primary mouse monoclonal anti-β-actin antibody (b, g, i). Double immunostainings of myc (green) with NR5A2 (red) in N2A cells (c-e, j-l). Arrows indicate double positive cells. Scale bars: 50 μm.

Supplementary Figure 19

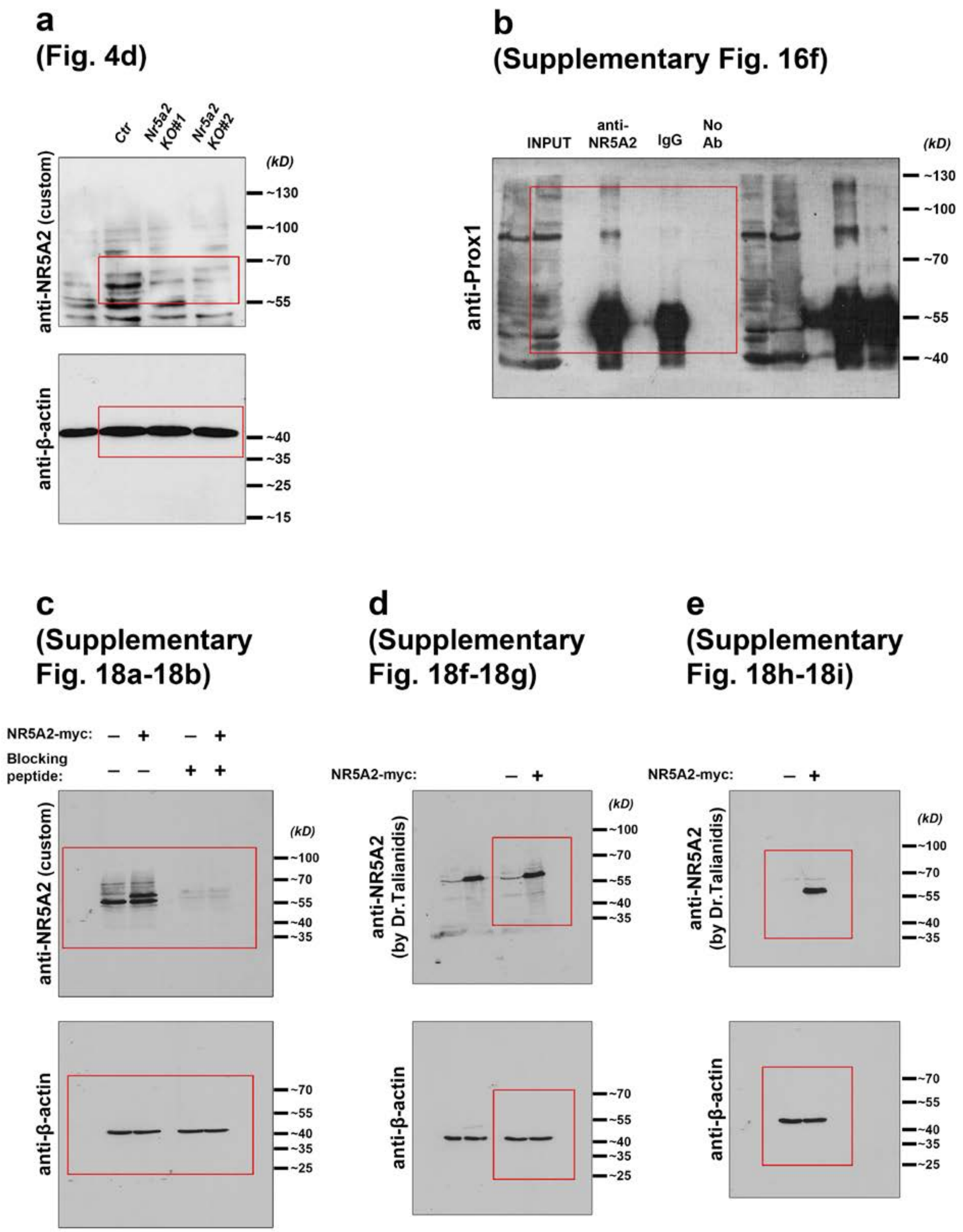

<u>Supplementary Figure 19</u>. Original full-size scans of the western blots presented in this study.

(a) Original scans of the western blots shown in Fig. 4d.

(b) Original scan of the western blot shown in Supplementary Fig. 16f.

(c-e) Original scans of the western blots shown in Supplementary Fig. 18.

Red boxes indicate the cropped regions of the western blots that were used in the figures. The numbers represent the molecular weights (kD). The antibody used is also shown.

Supplementary Table 1. Primer sets used in RT-qPCR assays.

Gene (mouse)	Forward qPCR Primer	Reverse qPCR Primer	*Reference*	Amplicon size (bp)
**Genes critical for induction of neuronal differentiation** (Fig. 6a)				
Brn2	CGGCGGTTTGCTCTATTC	ATGGTGTGGCTCATCGTG	*2*	118
Btg2	TGAGCGAGCAGAGACTCAAGGTTT	ACAGCGATAGCCAGAACCTTTGGA	*3*	113
Ctip2	GGGCGATGCCAGAATAGAT	GGTAGCCTCCACATGGTCAG	*4*	112
Cux1	TTGGCAAACAGGCAAGATGAGAGC	ACCTGCTTGCGTAAATCCTCTGGA	*5*	101
Cux2	CCTCAAGACGAACACCGTCAT	GCGCATCCTGGACCTGTAGT	*6*	55
Fezf1	CTGGCGACTCAGTCATGGA	GCAGTCGCTAGCATTTTGGT	*7*	61
Fezf2	GTGGCTCCCACCTTTGTACATTCA	TCACGGTGACAGGCTGGGATTAAA	*5*	121
NeuroD1	AGGAATTCGCCCACGCAGAA	TGGTCATGTTTCCACTTCCTGTTGT	*8*	101
Prox1	CAGCGGACTCTCTAGCACAG	GCCTGCCAAAAGGGGAAAGA	*1*	101
Satb2	GAGATGAGTTGAAGAGGGCTAGTG	CCCTGTGTGCGGTTGAAT	*7*	70
Sox11	CCCTGTCGCTGGTGGATAAG	GGTCGGAGAAGTTCGCCTC	*9*	138
Sox4	GAACGCCTTTATGGTGTGGT	GAACGGAATCTTGTCGCTGT	*9*	136
Tbr1	CCGAGTCCAGACGTTCACTT	GCCCGTGTAGATCGTGTCAT	*10*	142
Tbr2	TTCCGGGACAACTACGATTCA	ACGCCGTACCGACCTCC	*11*	56
**Genes involved in JAK/STAT pathway** (Fig. 9)				
Btk	GCTCTGTAGGCTCCAAGTTTC	ATCTCTCATACGGCATCTTCC	*12*	144
Cav1	GCGACCCCAAGCATCTCAA	ATGCCGTCGAAACTGTGTGT	*13*	91
Cntf	CTCTGTAGCCGCTCTATCTG	GGTACACCATCCACTGAGTC	*14*	125
Cntfr	ACAAGGTCTCCATAAGTGTCAGC	CTGCCATTGGTCCAGGATGA	*15*	236
Csf2rb	CTTCGCTTTGGCTGTGTCTCT	GACCTTTACCTCCATCCTGGAA	*16*	103
CT-1	GAGCCAGAGGGAGGGAAGTC	CAGCCGCGGTGGTGAGAAG	*17*	196
Eif2ak2	GTACAAGCGCTGGCAGAACTCAAT	AAGAGGCACCGGGTTTTGTAT	*18*	125
Hck	TGGATGGCCACCTACAACAA	CAGGAAGGCCTCCACGG	*19*	81
il10ra	TTCTCAGAACTAAAGAATGCAACCA	GGGCAGCACCTTGACACAA	*20*	99
il2rg	GGTTACTGAATACCAAGGGAACTTT	TGGCAGAACCGTTCACTGTA	*21*	96
il6ra	TGTTGAAGACTCTGCCAACCACGA	ACAGAGAAGCAACCCAAACGCCAA	*22*	148
irf9	CGCTGCTGCTCACCTTCAT	GGCTGTGCACCTGGGTCTTA	*23*	60
Jak1	GCCTGCCAGACTCCTGAC	CCTTCCAAAGCCCATTCA	*24*	95
Jak2	GATGGCGGTGTTAGACATGA	TGCTGAATGAATCTGCGAAA	*25*	177
Jak3	CTCCCCAGCGATTGTCATTCT	GCTCGCACACATAAATCCTCAG	*26*	61

Lif	ATGTGCGCCTAACATGACAG	TATGCGACCATCCGATACAG	27	90
Nlrc5	CTTCCCGCCTCTCCTTCCACAAT	CTCCACCTGCCCACATCCTACCA	28	161
Nmi	AGTGGAAAGCGTGGATTATGA	AATGCCTTCTAATCCGGTCA	29	226
Parp14	AGCAGTGGATCAGAAAAGACAG	GTCAGCACCATCTCGGATACT	30	107
Ptprc	GAGGTGTCTGATGGTGCAAG	TGTATTCCACTAAAGCCTGATGAA	31	65
Shp-1	GCAGGAGAACACTCGTGTCA	CCCATTGTCTAGTGGGGAGA	32	190
Stat1	CTGAATATTTCCCTCCTGGG	TCCCGTACAGATGTCCATGAT	33	103
Stat2	ATTGGAAGTTGCAGCGAGAG	TGCGCCATTTGGACTCTT	34	67
Stat3	ACCCAACAGCCGCCGTAG	CAGACTGGTTGTTTCCATTCAGAT	35	192
Stat4	TTCAGAGCAGCTCAACATGC	GGTGAGGTGACCATCATTGTAG	34	70
Stat5a	AAGATCAAGCTGGGGCACTA	CATGGGACAGCGGTCATAC	34	60
Stat5b	CGAGCTGGTCTTTCAAGTCA	CTGGCTGCCGTGAACAAT	34	64
Stat6	AGATGAGGCTTTCCGGAGTCA	CCCATATCTGAGCTGAGTTGCA	36	198
Tyk2	AGGGTCCCAAGCCTGAGTA	CTCAGGCAGTGCACTTAACC	37	150

<u>Cyclin-dependent kinase inhibitor (Cdki) and cell cycle-promoting genes</u>

(Fig. 8a-8b, Supplementary Fig. 3a,3d and Supplementary Fig. 17)

***Cdkn1a* (*p21*^{Cip1})**	GCAGACCAGCCTGACAGATT	CCTGACCCACAGCAGAAGAG	*designed*	108
***Cdkn1b* (*p27*^{Kip1})**	TCAGCGCAAGTGGAATTT	GGGCCTGTAGTAGAACTCG	38	106
***Cdkn2a-short variant* (*p16*^{Ink4a})**	GAACTCTTTCGGTCGTACCC	CAGTTCGAATCTGCACCGTA	*designed*	93
***Cdkn2a-short variant* (*p16*^{Ink4a})**	GAACTCTTTCGGTCGTACCC	CGAATCTGCACCGTAGTTGA	39	88
***Cdkn2a-short variant* (*p16*^{Ink4a})**	CCGCTGCAGACAGACTGG	CCATCATCATCACCTGAATCG	40	132
***Cdkn2a-long variant* (*p19*^{Arf})**	GCTCTGGCTTTCGTGAACAT	CGAATCTGCACCGTAGTTGA	*designed*	136
***Cdkn2b* (*p15*^{Ink4b})**	AGATCCCAACGCCCTGAAC	TCGTGCACAGGTCTGGTAAG	*designed*	144
Cyclin A	GCCTTCACCATTCATGTGGAT	TTGCTGCGGGTAAAGAGACAG	41	118
Cyclin D1	TAGGCCCTCAGCCTCACTC	CCACCCCTGGGATAAAGCA	*designed*	80
Cyclin	AAGCCCTCTGACCATTGTGTCC	CTAAGCAGCCAACATCCAGGAC	38	155

E1				
Ki67	CCTTTGCTGTCCCCGAAGA	GGCTTCTCATCTGTTGCTTCCT	*42*	104
Myc	CCACCAGCAGCGACTCTG	GTTTGCTGTGGCCTCGG	*43*	129
Nestin	CTGCAGGCCACTGAAAAGTT	TTCCAGGATCTGAGCGATCT	*designed*	90
**Genes encoding for TFs which are not directly related to neuronal differentiation** (Supplementary Fig. 12c)				
Atf3	AACTGGCTTCCTGTGCACTT	TGAGGCCAGCTAGGTCATCT	*44*	195
Hif1a	GGTTCCAGCAGACCCAGTTA	AGGCTCCTTGGATGAGCTTT	*45*	265
Klf10	GGGTGTGGCAAGACTTACTT	AATGGTCGCTCCTCATAAAC	*46*	208
Klf6	ACCAGACACTTCCGAAAGCA	TCTTAGCCTGGAAGCCTCTTT	*47*	161
Klf7	GAGCTCGGGGGAGGGTTTCTCC	GCCGGTGTCGTGGACAAGTTGT	*48*	107
Mef2a	AGCAGCACCATCTAGGACAA	CTGCTGTTGGAAGCCTGATG	*49*	164
Runx1	ACTTCCTCTGCTCCGTGCTA	CGCGGTAGCATTTCTCAGTT	*50*	161
**Genes critical for astrocyte development** (Supplementary Fig. 3b, 3e and Supplementary Fig. 9)				
Aldh1l1	ATTCCCAAGGGTGTGGTCAAC	CATCAGGGTGGTCTGAGAGTCTCT	*51*	76
Cd44	ATCAGCAGATCGATTTGAATGTAA	CATTTCCTTCTATGAACCCATACC	*52*	201
Fabp7	CTCTGGGCGTGGGCTTT	TTCCTGACTGATAATCACAGTTGGTT	*53*	71
Fgfr3	GGCCCTTTTTGACCGAGTCTA	CCCAGCGTAAAGATCTCCCAG	*51*	81
Gfap	GAAAGGTTGAATCGCTGGAG	CACGTGGACCTGCTGTTG	*designed*	104
Gja1	ATGCTGGTGGTGTCCTTGGTG	CGGTGGTGGCGTGGTAAGG	*54*	118
Id3	CACTTACCCTGAACTCAACGCC	CCCATTCTCGGAAAAGCCAG	*12*	162
Nfia	AAGCCTCCAACCACATCAAC	TTTACAAAGCTTGGATCCCG	*http://scholarworks.gvsu.edu/sss/8*	125
Nfib	GAGGTCGCTGTCTTCTCCAC	ACAGTGGCTTCTCAGGCTTC	*55*	191
S100β	TGGTTGCCCTCATTGATGTCT	CCCATCCCCATCTTCGTCC	*designed*	179
Slc1a3	GAAGCCATCATGCGATTGG	CAACAATCTTCCCTGCGATCA	*51*	82
**Genes used as extra internal references (housekeeping genes)** (Supplementary Fig. 12a-12b)				
Ppia	CGCGTCTCCTTCGAGCTGTTTG	TGTAAAGTCACCACCCTGGCACAT	*56*	150
Rpl13a	TTCGGCTGAAGCCTACCAGAAAGT	TCTTCCGATAGTGCATCTTGGCCT	*57*	132
***Nr5a2* and *Prox1* primers to avoid contamination with overexpression vectors**				
Nr5a2-avoid	GCATGCCAAAAGAGCCTAAG	CATTGCTTGGAGCAGTTCAG	*designed*	116
Prox1-	GAGCTTTTGAAGATGGCACA	TCCAATGTCAACCCTTCTCC	*designed*	100

avoid				
**Other genes**				
Cre	GATTTCGACCAGGTTCGTTC	GCTAACCAGCGTTTTCGTTC	*www.ebiomethods.com*	195
Dcx	ATGCAGTTGTCCCTCCATTC	ATGCCACCAAGTTGTCATCA	[58]	182
Map2	GCTGAAGCTGTAGCAGTCCTGAA	GTGTTGGGCTTCCTTCTCTTGT	[59]	51
NeuN (Rbfox3)	GGCAAATGTTCGGGCAATTCG	TCAATTTTCCGTCCCTCTACGAT	*designed*	160
Sox2	GCGGAGTGGAAACTTTTGTCC	GGGAAGCGTGTACTTATCCTTCT	*designed*	156
βIII-tubulin (Tubb3)	CCTGGAACCATGGACAGTGTT	CAGCACCACTCTGACCAAAGA	[60]	85

Supplementary References

1. Kaltezioti V, *et al.* Prox1 regulates the notch1-mediated inhibition of neurogenesis. *PLoS biology* **8**, e1000565 (2010).

2. Goodall J, *et al.* Brn-2 represses microphthalmia-associated transcription factor expression and marks a distinct subpopulation of microphthalmia-associated transcription factor-negative melanoma cells. *Cancer research* **68**, 7788-7794 (2008).

3. Terra R, Luo H, Qiao X, Wu J. Tissue-specific expression of B-cell translocation gene 2 (BTG2) and its function in T-cell immune responses in a transgenic mouse model. *International immunology* **20**, 317-326 (2008).

4. Tydell CC, David-Fung ES, Moore JE, Rowen L, Taghon T, Rothenberg EV. Molecular dissection of prethymic progenitor entry into the T lymphocyte developmental pathway. *J Immunol* **179**, 421-438 (2007).

5. Wang ZB, *et al.* Fezf2 regulates telencephalic precursor differentiation from mouse embryonic stem cells. *Cereb Cortex* **21**, 2177-2186 (2011).

6. Conforto TL, Zhang Y, Sherman J, Waxman DJ. Impact of CUX2 on the female mouse liver transcriptome: activation of female-biased genes and repression of male-biased genes. *Molecular and cellular biology* **32**, 4611-4627 (2012).

7. Kurrasch DM, Cheung CC, Lee FY, Tran PV, Hata K, Ingraham HA. The neonatal ventromedial hypothalamus transcriptome reveals novel markers with spatially distinct patterning. *The Journal of neuroscience : the official journal of the Society for Neuroscience* **27**, 13624-13634 (2007).

8. Mwangi SM, *et al.* Glial cell line-derived neurotrophic factor enhances neurogenin3 gene expression and beta-cell proliferation in the developing mouse pancreas. *American journal of physiology Gastrointestinal and liver physiology* **299**, G283-292 (2010).

9. Mu L, *et al.* SoxC transcription factors are required for neuronal differentiation in adult hippocampal neurogenesis. *The Journal of neuroscience : the official journal of the Society for Neuroscience* **32**, 3067-3080 (2012).

10. Buttner N, Johnsen SA, Kugler S, Vogel T. Af9/Mllt3 interferes with Tbr1 expression through epigenetic modification of histone H3K79 during development of the cerebral cortex. *Proceedings of the National Academy of Sciences of the United States of America* **107**, 7042-7047 (2010).

11. Fukuyama T, Kasper LH, Boussouar F, Jeevan T, van Deursen J, Brindle PK. Histone acetyltransferase CBP is vital to demarcate conventional and innate CD8+ T-cell development. *Molecular and cellular biology* **29**, 3894-3904 (2009).

12. Sokalski KM, Li SK, Welch I, Cadieux-Pitre HA, Gruca MR, DeKoter RP. Deletion of genes encoding PU.1 and Spi-B in B cells impairs differentiation and induces pre-B cell acute lymphoblastic leukemia. *Blood* **118**, 2801-2808 (2011).

13. Shostak A, Meyer-Kovac J, Oster H. Circadian regulation of lipid mobilization in white adipose tissues. *Diabetes* **62**, 2195-2203 (2013).

14. Agca C, *et al.* p38 MAPK signaling acts upstream of LIF-dependent neuroprotection during photoreceptor degeneration. *Cell death & disease* **4**, e785 (2013).

15. McGregor NE, *et al.* Ciliary neurotrophic factor inhibits bone formation and plays a sex-specific role in bone growth and remodeling. *Calcified tissue international* **86**, 261-270 (2010).

16. Kappen C, Kruger C, MacGowan J, Salbaum JM. Maternal diet modulates placenta growth and gene expression in a mouse model of diabetic pregnancy. *PloS one* **7**, e38445 (2012).

17. Marques JM, Belza I, Holtmann B, Pennica D, Prieto J, Bustos M. Cardiotrophin-1 is an essential factor in the natural defense of the liver against apoptosis. *Hepatology* **45**, 639-648 (2007).

18. Stobart MJ, *et al.* Differential expression of interferon responsive genes in rodent models of transmissible spongiform encephalopathy disease. *Molecular neurodegeneration* **2**, 5 (2007).

19. Parisi S, *et al.* Direct targets of Klf5 transcription factor contribute to the maintenance of mouse embryonic stem cell undifferentiated state. *BMC biology* **8**, 128 (2010).

20. Ueda Y, *et al.* Commensal microbiota induce LPS hyporesponsiveness in colonic macrophages via the production of IL-10. *International immunology* **22**, 953-962 (2010).

21. Kerdiles YM, *et al.* Foxo1 links homing and survival of naive T cells by regulating L-selectin, CCR7 and interleukin 7 receptor. *Nature immunology* **10**, 176-184 (2009).

22. Pieraut S, *et al.* An autocrine neuronal interleukin-6 loop mediates chloride accumulation and NKCC1 phosphorylation in axotomized sensory neurons. *The Journal of neuroscience : the official journal of the Society for Neuroscience* **31**, 13516-13526 (2011).

23. Kamei Y, *et al.* Increased expression of DNA methyltransferase 3a in obese adipose tissue: studies with transgenic mice. *Obesity (Silver Spring)* **18**, 314-321 (2010).

24. Li Y, *et al.* Inflammatory signaling regulates embryonic hematopoietic stem and progenitor cell production. *Genes & development* **28**, 2597-2612 (2014).

25. Koppikar P, *et al.* Heterodimeric JAK-STAT activation as a mechanism of persistence to JAK2 inhibitor therapy. *Nature* **489**, 155-159 (2012).

26. Murakami M, *et al.* Disease-association analysis of an inflammation-related feedback loop. *Cell reports* **3**, 946-959 (2013).

27. Moidunny S, *et al.* Adenosine A2B receptor-mediated leukemia inhibitory factor release from astrocytes protects cortical neurons against excitotoxicity. *Journal of neuroinflammation* **9**, 198 (2012).

28. Biswas A, Meissner TB, Kawai T, Kobayashi KS. Cutting edge: impaired MHC class I expression in mice deficient for Nlrc5/class I transactivator. *J Immunol* **189**, 516-520 (2012).

29. Pfeffer LM, *et al.* Role of nuclear factor-kappaB in the antiviral action of interferon and interferon-regulated gene expression. *The Journal of biological chemistry* **279**, 31304-31311 (2004).

30. Wang Y, Inger M, Jiang H, Tenenbaum H, Glogauer M. CD109 plays a role in osteoclastogenesis. *PloS one* **8**, e61213 (2013).

31. Takase HM, *et al.* FGF7 is a functional niche signal required for stimulation of adult liver progenitor cells that support liver regeneration. *Genes & development* **27**, 169-181 (2013).

32. Casey SC, Blumberg B. The steroid and xenobiotic receptor negatively regulates B-1 cell development in the fetal liver. *Mol Endocrinol* **26**, 916-925 (2012).

33. Shaul ME, Bennett G, Strissel KJ, Greenberg AS, Obin MS. Dynamic, M2-like remodeling phenotypes of CD11c+ adipose tissue macrophages during high-fat diet--induced obesity in mice. *Diabetes* **59**, 1171-1181 (2010).

34. Lange C, Thiersch M, Samardzija M, Grimm C. The differential role of Jak/STAT signaling in retinal degeneration. *Advances in experimental medicine and biology* **664**, 601-607 (2010).

35. Mechoulam H, Pierce EA. Expression and activation of STAT3 in ischemia-induced retinopathy. *Investigative ophthalmology & visual science* **46**, 4409-4416 (2005).

36. Hart GT, *et al.* Quantitative gene expression profiling implicates genes for susceptibility and resistance to alveolar bone loss. *Infection and immunity* **72**, 4471-4479 (2004).

37. Niederreiter L, *et al.* ER stress transcription factor Xbp1 suppresses intestinal tumorigenesis and directs intestinal stem cells. *The Journal of experimental medicine* **210**, 2041-2056 (2013).

38. Foskolou IP, Stellas D, Rozani I, Lavigne MD, Politis PK. Prox1 suppresses the proliferation of neuroblastoma cells via a dual action in p27-Kip1 and Cdc25A. *Oncogene* **32**, 947-960 (2013).

39. He J, Nguyen AT, Zhang Y. KDM2b/JHDM1b, an H3K36me2-specific demethylase, is required for initiation and maintenance of acute myeloid leukemia. *Blood* **117**, 3869-3880 (2011).

40. Hacker E, *et al.* Spontaneous and UV radiation-induced multiple metastatic melanomas in Cdk4R24C/R24C/TPras mice. *Cancer research* **66**, 2946-2952 (2006).

41. Clark SP, Davis MA, Ryan TP, Searfoss GH, Hooser SB. Hepatic gene expression changes in mice associated with prolonged sublethal microcystin exposure. *Toxicologic pathology* **35**, 594-605 (2007).

42. Huang HP, *et al.* Epithelial cell adhesion molecule (EpCAM) complex proteins promote transcription factor-mediated pluripotency reprogramming. *The Journal of biological chemistry* **286**, 33520-33532 (2011).

43. Stellas D, *et al.* Therapeutic effects of an anti-Myc drug on mouse pancreatic cancer. *Journal of the National Cancer Institute* **106**, (2014).

44. Yoshida T, *et al.* ATF3 protects against renal ischemia-reperfusion injury. *Journal of the American Society of Nephrology : JASN* **19**, 217-224 (2008).

45. Nytko KJ, Maeda N, Schlafli P, Spielmann P, Wenger RH, Stiehl DP. Vitamin C is dispensable for oxygen sensing in vivo. *Blood* **117**, 5485-5493 (2011).

46. Zhang W, *et al.* Klf10 inhibits IL-12p40 production in macrophage colony-stimulating factor-induced mouse bone marrow-derived macrophages. *European journal of immunology* **43**, 258-269 (2013).

47. Ueharu H, *et al.* Expression of Kruppel-like factor 6, KLF6, in rat pituitary stem/progenitor cells and its regulation of the PRRX2 gene. *The Journal of reproduction and development* **60**, 304-311 (2014).

48. Guo T, Mandai K, Condie BG, Wickramasinghe SR, Capecchi MR, Ginty DD. An evolving NGF-Hoxd1 signaling pathway mediates development of divergent neural circuits in vertebrates. *Nature neuroscience* **14**, 31-36 (2011).

49. Escher P, Schorderet DF, Cottet S. Altered expression of the transcription factor Mef2c during retinal degeneration in Rpe65-/- mice. *Investigative ophthalmology & visual science* **52**, 5933-5940 (2011).

50. Knezevic K, *et al.* A Runx1-Smad6 rheostat controls Runx1 activity during embryonic hematopoiesis. *Molecular and cellular biology* **31**, 2817-2826 (2011).

51. Kamphuis W, *et al.* GFAP isoforms in adult mouse brain with a focus on neurogenic astrocytes and reactive astrogliosis in mouse models of Alzheimer disease. *PloS one* **7**, e42823 (2012).

52. Huebener P, *et al.* CD44 is critically involved in infarct healing by regulating the inflammatory and fibrotic response. *J Immunol* **180**, 2625-2633 (2008).

53. Gerstner JR, Bremer QZ, Vander Heyden WM, Lavaute TM, Yin JC, Landry CF. Brain fatty acid binding protein (Fabp7) is diurnally regulated in astrocytes and hippocampal granule cell precursors in adult rodent brain. *PloS one* **3**, e1631 (2008).

54. Gunther S, Fietz D, Weider K, Bergmann M, Brehm R. Effects of a murine germ cell-specific knockout of Connexin 43 on Connexin expression in testis and fertility. *Transgenic research* **22**, 631-641 (2013).

55. Tsuyama J*, et al.* MicroRNA-153 Regulates the Acquisition of Gliogenic Competence by Neural Stem Cells. *Stem cell reports* **5**, 365-377 (2015).

56. Mamo S, Gal AB, Bodo S, Dinnyes A. Quantitative evaluation and selection of reference genes in mouse oocytes and embryos cultured in vivo and in vitro. *BMC developmental biology* **7**, 14 (2007).

57. Leystra AA*, et al.* Mice expressing activated PI3K rapidly develop advanced colon cancer. *Cancer research* **72**, 2931-2936 (2012).

58. Santra M, Santra S, Buller B, Santra K, Nallani A, Chopp M. Effect of doublecortin on self-renewal and differentiation in brain tumor stem cells. *Cancer science* **102**, 1350-1357 (2011).

59. Fischer B, Azim K, Hurtado-Chong A, Ramelli S, Fernandez M, Raineteau O. E-proteins orchestrate the progression of neural stem cell differentiation in the postnatal forebrain. *Neural development* **9**, 23 (2014).

60. Ringhoff DN, Cassimeris L. Gene expression profiles in mouse embryo fibroblasts lacking stathmin, a microtubule regulatory protein, reveal changes in the expression of genes contributing to cell motility. *BMC genomics* **10**, 343 (2009).
